# Perceptions of the rabbit as a low investment ‘starter pet’ lead to negative impacts on its welfare: Results of two Danish surveys

**DOI:** 10.1017/awf.2023.41

**Published:** 2023-07-03

**Authors:** Cecilie Ravn Skovlund, Björn Forkman, Thomas Bøker Lund, Belinda Glumsøe Mistry, Søren Saxmose Nielsen, Peter Sandøe

**Affiliations:** 1 Department of Veterinary and Animal Sciences, Faculty of Health and Medical Sciences, University of Copenhagen, Grønnegårdsvej 15, 1870 Frederiksberg C, Denmark; 2 Department of Food and Resource Economics, Faculty of Science, University of Copenhagen, Rolighedsvej 23, 1958, Frederiksberg C, Denmark

**Keywords:** Animal welfare, companion animal, husbandry, owner perception, rabbit, survey

## Abstract

Concerns over compromised companion rabbit (*Oryctolagus cuniculus domesticus*) welfare are widespread. The welfare problems have been linked to the perception of rabbits as low investment ‘children’s pets.’ To test this hypothesis and investigate the current conditions for rabbits, data were gathered from two surveys in 2021: a nationally representative survey of Danish companion animal owners (Survey I) and a detailed social media-based survey of Danish rabbit owners (Survey II). Using logistic regression, three owner-related variables (whether a child/adult was responsible for care of the rabbit, owner-opinion on rabbits’ suitability as ‘starter pets’ and willingness-to-pay [WTP] for veterinary treatment) were employed to investigate the effect of rabbit status on owner-provision of selected husbandry conditions. The 76 (Survey I) and 4,335 (Survey II) responses suggested that most rabbits are acquired for children and are solitarily housed, and that many are kept in cages of an unsuitable size and not checked daily. Owners who perceived rabbits as ‘starter pets’ and with lower WTP were more likely to house rabbits in restricted space and to not provide continuous gnawing opportunities, *ad libitum* hay or routine healthcare. A child fulfilling the role of the rabbit’s main caretaker was also associated with inadequate housing type and fewer gnawing opportunities. Thus, many rabbits live in unsuitable conditions, and owners who perceive rabbits as low investment ‘children’s pets’ are more likely to not provide recommended resources. Changing owners’ perceptions of rabbits and promoting suitable husbandry through official education programmes and minimum requirements is important if there are to be improvements made to rabbit welfare.

## Introduction

The domestic rabbit (*Oryctolagus cuniculus domesticus*) is kept for various purposes, including experimental research, meat production and showing in exhibitions, but in Denmark and most other European countries its most common role is as a companion animal. It has been ranked as the third most popular companion animal after dogs and cats in various European countries (Schepers *et al.* 2009; Mäkitaipale *et al.* 2015; Ulfsdotter *et al.* 2016; Pet Food Manufacturers’ Association [PFMA] 2019; Mee *et al.* 2022; People’s Dispensary for Sick Animals [PDSA] 2022). Its wild counterpart lives in large colonies made up of several stable groups (DiVincenti & Rehrig 2016) with large individual territory sizes (Surridge *et al.* 1999; Devillard *et al.* 2008; d’Ovidio *et al.* 2016). In contrast, domestic rabbits tend to live in very different conditions, including restricted space allowance, solitary housing, imposed group formations, high stocking density and with limited behavioural opportunities (e.g. Seaman *et al.* 2008; Tschudin *et al.* 2011; Andrist *et al.* 2012; Stapleton 2014; DiVincenti & Rehrig 2016; El-Sabrout 2018; Windschnurer *et al.* 2019; Dalmau *et al.* 2020).

There is a wealth of research into the welfare effects of the husbandry conditions of rabbits kept for meat production and research. Connected with this, legislative initiatives and recommendations on minimum requirements have been developed (e.g. European Food Safety Authority [EFSA] panel 2020; Danish Ministerial Order regarding Animal Experimentation 2021). For companion rabbits, however, there are limited legal requirements (Rioja-Lang *et al.* 2019; Dixon 2021), and despite their popularity as companion animals and, probably, wide variation in how they are kept, little research has been undertaken into the housing and husbandry conditions of companion rabbits. The few studies that have been carried out show that many companion rabbits are managed under inadequate husbandry, consequently raising concerns about their welfare. For example, health problems, unsuitable feeding regimes and estimates of reduced lifespan have been reported in companion rabbits in several European countries (Schepers *et al.* 2009; Stapleton 2014; Mäkitaipale *et al.* 2015; Johnson & Burn 2019; O’Neill *et al.* 2020), as have various less than optimal husbandry conditions, including housing rabbits solitarily or under inappropriate social housing arrangements, incorporating little or no enrichment, using unsuitable sized housing and inappropriate handling methods, among other welfare challenges (Mullan & Main 2006; Schepers *et al.* 2009; Edgar & Mullan 2011; Rooney *et al.* 2014; McIndoe *et al.* 2022; Mee *et al.* 2022; PDSA 2022). All of these husbandry conditions have been highlighted by experts as seriously impinging on the welfare of rabbits (Rioja-Lang *et al.* 2019).

An important factor explaining the high level of occurrence of unsuitable husbandry conditions may be the common owner-perception that the rabbit is a low-investment ‘children’s pet’ (Mullan & Main 2006). It has been argued that the prevailing view of the rabbit as a companion animal that is suitable mainly for children and as a ‘starter pet’ poses a risk of inadequate care (Rioja-Lang *et al.* 2019; PDSA 2020). The view may also have led to misconceptions about the rabbit’s lifespan and husbandry requirements, ultimately contributing to the large numbers of rabbits relinquished at shelters each year (Cook & McCobb 2012; Ulfsdotter *et al.* 2016; Ellis *et al.* 2017; Neville *et al.* 2019). Coupled with a general lack of owner knowledge regarding rabbits’ needs, which has already been found to increase the risk of inadequate husbandry, owners’ perceptions of rabbits may negatively affect rabbit welfare on several levels (Edgar & Mullan 2011; Welch *et al.* 2017; Rioja-Lang *et al.* 2019). It has therefore been suggested that targeting owners’ perceptions of rabbits is a vital step towards improving companion rabbit welfare (Edgar & Mullan 2011; McMahon & Wigham 2020).

Despite the reported concerns regarding the welfare of companion rabbits, there is little information on the conditions under which they are actually kept. The aim of this study was therefore to investigate these conditions in relation to existing recommendations for rabbit husbandry, and to identify areas of potential concern in terms of welfare. Moreover, since it appears that rabbits are at risk of receiving inadequate care as a result of their reported status as low-investment ‘children’s pets’ and ‘starter pets’, the study also aimed to investigate whether owner-related factors relating to perceptions about the status of companion rabbits affect their welfare.

We consider the following factors to be potential indicators of low rabbit status as perceived by owners: that a child is responsible for the care of the rabbit; that the rabbit is viewed as a suitable ‘starter pet’ for children; and that the owner has a low willingness-to-pay (WTP) for life-saving veterinary treatment (based on a hypothetical scenario about WTP for such treatment). We assessed rabbit welfare through a number of rabbit husbandry conditions (set out in detail later: see *Materials and methods, Survey II*). We hypothesised that the owner-related factors mentioned above would be associated with an increased likelihood of husbandry conditions that are identified in the literature as risks to rabbit welfare. The husbandry conditions and owner perceptions of companion rabbits were investigated in two surveys of rabbit owners in Denmark: one (Survey I) provided nationally representative data on the prevalence of the different approaches to rabbit housing employed by companion rabbits owners in Denmark; the other (Survey II), which was a larger web-based survey, provided detailed information on rabbit husbandry identified as being of importance to rabbits’ needs.

## Materials and methods

### Data collection

#### Survey I

##### Survey I data and materials

Survey I was part of a larger project investigating the distribution of companion animals in Denmark and owner-companion animal attachment. It involved a cross-sectional questionnaire and was carried out by Statistics Denmark using a random sample of Danish households. In all, 5,027 people were drawn from the Danish Central Personal Registry and invited to participate in the web-based survey via email (Statistics Denmark have permission to send emails to all Danish households except those exempted). Invitees who did not respond to the emailed invitation were contacted by telephone, or letter, and encouraged to participate. Survey responses were collected between May and June 2021. The questions (in Danish), which were aimed at companion rabbit owners, were designed to provide insights into the distribution of companion animal rabbits and general information about the conditions the rabbits were housed under, including housing type (cage, run or free-roam housing), indoor/outdoor housing, purpose of acquisition, and daily access to various resources. Details of the Survey I questions can be found in the Supplementary materials (where the questions have been translated into English). Respondents were asked to enter information for a single rabbit in their household. If there were more than one, they were asked to order the rabbits’ names alphabetically and enter information for the first rabbit only.

##### Survey I data analysis

Frequencies and percentages were used in the presentation of the results to represent the proportion of households with companion rabbits. To ensure the results were representative, weighted proportions are reported using a weight variable that adjusts the sample so that it matches the background population of approximately 3.1 million families in Denmark. The weight variable was constructed by Statistics Denmark based on the following census variables at family level: population density; family income; number of family members; region of Denmark; housing type; dwelling size; and family type (i.e. ‘single without children’, ‘single with children’, ‘couple with children’, and ‘couple without children’).

#### Survey II

Survey II was part of a separate project (Skovlund *et al.* 2022) and was distributed to Danish companion rabbit owners. It was a cross-sectional, web-based questionnaire study using convenience sampling (i.e. there was not a probability-based recruitment principle: see recruitment details below). The questionnaire contained rabbit-related questions (in Danish) similar to those used in Survey I together with supplementary and more in-depth questions designed to elicit information on the rabbits, their owners and husbandry conditions.

##### Survey II data and materials

Survey II was developed in SurveyXact (Rambøll Management Consulting, Aarhus N, Denmark) and distributed online, mainly through Facebook and Facebook groups, for six weeks in the period September–November 2021. The survey was open to persons ≥ 18 years owning companion rabbits at the time of participating in the survey. The survey was distributed mainly through a hyperlink shared on a Facebook page dedicated to the project. To reach a wider range of respondents two Facebook advertisements were created: one was aimed at all Danish Facebook account holders and the other at Danish parents (≥ 18 years). The rationale for the second advertisement was that rabbits are often acquired for children, and that these children would not be completing the survey owing to the age limit. The survey was also shared in various rabbit- and non-rabbit-related Facebook groups.

The survey questions and response options were based on rabbit literature and welfare concerns commonly reported for companion rabbits. Common response options were identified by piloting the questions (open-question style) to four rabbit owners. The final survey was piloted on four other rabbit owners. A mix of mandatory and optional questions were included in it, as well as both single- and multiple-choice questions (allowing multiple responses) in which the order of response options was randomised for each respondent. As in Survey I, respondents with more than one rabbit entered information for the rabbit whose name came first alphabetically. The subset of the survey utilised in this study included questions on owner demographics, rabbit information (breed, sex, age and neutering status), and husbandry conditions (including housing, social environment, diet, resource and healthcare provision, such as veterinary visits). To assess the average lifespan of companion rabbits in Denmark, we also asked respondents to provide the age of their most recently deceased rabbit. Owners keeping their rabbits in a cage were presented with optional questions about the cage’s dimensions, as well as about the daily time and length of period the rabbit was given outside-cage access. Furthermore, to understand owners’ husbandry decisions that had been identified as having important implications for rabbit welfare, questions regarding the reasoning behind those decisions were included as optional multiple-choice questions, and these were supplemented with open-ended response options to obtain additional information. Respondents were finally asked questions about their view of the value, and the status, of rabbits as companion animals. Here, the questions were about WTP for life-saving veterinary treatment (reflecting investment and monetary value), whether a child or adult was responsible for the rabbit’s care, and who the rabbit had been acquired for, as well as owner-attitudes as to the suitability of rabbits as companion animals for owners at different life-stages (specifically in childhood and adulthood). Details of the Survey II questions (translated into English) can be found in Supplementary materials.

##### Survey II data analysis

###### Descriptive data

The survey included several measures which are presented using frequencies and percentages. (Frequencies are provided for all the variables used in the regression analyses). The following is an overview of these measures.

Space available to the rabbit was assessed through reported housing dimensions, including cage length, width, height (all in m) and estimated total area (m^2^). (A few cases of dimensions incorrectly reported in cm [instead of m] were identified and adjusted accordingly). Stocking density was calculated from the reported number of rabbits in the provided space (m^2^). Housing dimensions were compared with the requirements laid down in Danish regulations on the housing of laboratory rabbits, using the minimum requirements for rabbits older than ten weeks and of < 3 kg bodyweight as the reference, as this applies to the most commonly kept companion rabbit breeds in this study (see *Results* for the breeds that were reported in Survey II): total area: 3.5 m^2^; enclosure height: 0.45 m; stocking density: 0.57 rabbit m^–2^ (1–2 rabbits allowed on 3.5 m^2^; calculated as 2/3.5 m^2^) (Danish Ministerial Order regarding Animal Experimentation 2021). No regulations on enclosure length and width were found for laboratory rabbits. Therefore, here, the recommendations issued by Animal Protection Denmark (Dyrenes Beskyttelse: DB) were used instead (length: 1.20 m; width: 0.8 m; Dyrenes Beskyttelse undated).

All of the open-ended responses regarding owners’ reasons for choices of rabbit husbandry were recoded into novel overarching categories by a single rater (BGM) (e.g. ‘Why do you house your rabbit alone?’ resulted in the overarching category ‘I was told by the breeder/pet shop that rabbits should be housed alone’) (Table S3). The open-ended responses were assigned into overarching categories by manually grouping them into topics, when ≥ 10 respondents reported the same reason that was not already present in the original multiple-choice options. The new categories were interpreted and labelled in consultation with CRS. When an open response belonged to one of the existing closed options, the respondent was assigned the value of the corresponding closed option. After the process of recoding (BGM) and developing overarching categories (BGM and CRS), 50 responses for each overarching category were chosen randomly and used to analyse inter-rater reliability. Since BGM and CRS had been involved in discussing the overarching categories, inter-rater reliability was based on recoded responses of an additional independent rater from outside the research group, with knowledge on companion rabbit husbandry. Therefore, three raters (HW, BGM and CRS) were employed in total. We used Fleiss’ kappa statistics, and 0.60 ≥ κ was deemed adequate agreement (fulfilled for all the overarching categories).

###### Uni- and multivariable analysis

Binary logistic regression models were used to analyse whether rabbit status, as perceived by owners (owner variables), were associated with husbandry conditions (outcome variables).

###### Outcome variables

The outcome variables were based on a number of resource and management indicators deemed to be relevant to animal welfare. The values of these indicators were set as dichotomous values corresponding to ‘adequate’ and ‘inadequate’ conditions for the purpose of testing the hypotheses. The variables and their relevance to rabbit welfare were identified through literature and existing recommendations and legislation on rabbit husbandry. To cover multiple aspects of welfare, the variables represented each of the Five Welfare Needs (companionship, housing, behaviour/resources, diet and health) laid down for companion rabbits in response to the Animal Welfare Act (2006) of the United Kingdom (as applied in England, Scotland and Wales only) (e.g. in Welsh Government 2009; DAERA 2011). Variables with sufficient data-points per category (≥ 20) across owner variables were included for analysis. The variables were merged into six dichotomous variables: ‘Social Housing’ (Solitary vs Social); ‘Housing Type’ (spatial restriction: Cage or run housed vs Free or partly free roam); ‘Space Availability’ (total area [m^2^] relative to requirements for Danish laboratory rabbits: Below vs Above); ‘Resource Provision’ (opportunity to gnaw: Continuous opportunity vs No continuous opportunity); ‘Diet’ (provision of hay/grass: *Ad libitum* vs Not *ad libitum*) and ‘Veterinary Access’ (regular annual visits: Not Regular vs Regular). Besides relating to each of the Five Welfare Needs, these variables were chosen in response to recommendations and requirements that rabbits should: (1) be housed with conspecifics; (2) have limited behavioural and physical restriction; (3) be provided with the opportunity to gnaw; (4) have unlimited access to hay or grass; and (5) have at least annual health check-ups by a veterinarian (e.g. Dixon *et al.* 2010; Buijs *et al.* 2011; Clauss & Hatt 2017; Rioja-Lang *et al.* 2019; All-Party Parliamentary Group for Animal Welfare [APGAW] 2021). We created values that were dichotomous because there were few data-points per category and for ease of interpretation (pre-coded categories can be seen in Supplementary materials; Table S1).

###### Owner variables

Three owner variables were used to investigate the potential effects of the perceived status of rabbits on husbandry conditions. These included ‘Responsibility’ (whether a child < 18 years or an adult ≥ 18 years was the main person responsible for rabbit care in the household, ‘Starter Pet’ (the owner’s opinion on suitability of rabbits as a child’s first-time companion animal) and ‘WTP’ (for veterinary treatment). Owners indicating low WTP were interpreted as people perceiving the rabbit to be an animal requiring a low level of investment and of low monetary value. Based on descriptive statistics (Table S4; Supplementary materials) revealing few observations per category, WTP and Responsibility were collapsed into fewer categories (categories of WTP in the model of Space Availability were further collapsed owing to few data-points per level: see Table 2).

**Table 1.** Parameters of dimensions of housing for companion rabbits, and comparison with minimum requirements for Danish laboratory rabbits and the recommendations of Animal Protection Denmark

Parameter	*n*	Median	Min.	IQR	Did not meet requirements/recommendations *LAB ^1^ = laboratory rabbits, DB ^2^ = Animal Protection Denmark*
*Cage*					
Length (m)	923	1.5	0.5	1.2–2	19% (172) ^DB^
Width (m)	914	0.8	0.3	0.6–1.2	44% (398) ^DB^
Height (m)^b^	374	0.65	0.3	0.5–1	9% (34) ^LAB^
Area (m)^2^	589	2	0.2	1–4	68% (399) ^LAB^
No of storeys	885	2	0.5	1–2	–
No of rabbits in cage	326	2	1	2–2	–
Stocking density, rabbit m^–2 a^	187	0.5	0.03	0.30-1	47% (87) ^LAB^
*Semi-free/free-roam*					
Area (m^2^)	2,893	40	0.5	12–80	3% (93) ^LAB^
No. of rabbits in area	1,200	2	1	2–2	–
Stocking density rabbit m^–2 a^	1,132	0.08	0	0.03–0.2	3% (29) ^LAB^

Abbreviations: Min, Minimum; IQR, Interquartile Range

Empty ( - ): No identified requirements/recommendations.

a Stocking density subsequently calculated based on data from responses.

b Only includes heights for single-storey cages, since height for cages with several storeys may not depict the actual height that is available to the rabbit(s).

1 Minimum requirements for laboratory rabbits (requirements used for rabbits over 10 weeks of < 3 kg body weight): M^2^ = 3.5; Height, m = 0.45; stocking density = 0.57 (1–2 rabbits allowed on 3.5, thus 2/3.5 m^2^) (Danish Ministerial Order regarding Animal Experimentation 2021).

2 Recommendations, DB: Length, m = 1.20; Width, m = 0.8 (Dyrenes beskyttelse n.d.).

**Table 2.** Univariable logistic analyses of owner variables associated with rabbit husbandry conditions (n = 4,335)

		Social Housing				Housing Type			
Owner variable	Category	*Social n (%)*	*Solitary n (%)*	Odds ratio (95% CI)	*P-value*	*Free roam (full or partly) n (%)*	*Cage or Run n (%)*	Odds ratio (95% CI)	*P-value*
Responsibility		1,521 (35%)	2,784 (65%)		0.449	2,643	1,662		**<0.001**
	Adult	1,049 (69%)	1,897 (68%)	1 (ref)		1,883 (71%)	1,063 (64%)	1 (ref)	
	All, equally	373 (25%)	677 (24%)	1.0 (0.9–1.2)		669 (25%)	381 (23%)	1.0 (0.9–1.2)	
	Child	99 (7%)	210 (8%)	1.2 (0.9–1.5)		91 (3%)	218 (13%)	4.2 (3.3–5.5)	
Starter Pet		1,503 (35%)	2,748 (65%)		**0.093**	2,611	1,640		**<0.001**
	Disagree	574 (38%)	964 (34%)	1 (ref)		1,183 (453%)	355 (22%)	1 (ref)	
	Partly disagree	273 (18%)	530 (19%)	1.2 (1.0–1.4)		510 (20%)	293 (18%)	1.9 (1.6–2.3)	
	Partly agree	406 (27%)	731 (26%)	1.1 (0.9–1.3)		586 (22%)	551 (34%)	3.1 (2.7–3.7)	
	Agree	250 (16%)	523 (17%)	1.3 (1.0–1.5)		332 (13%)	441 (27%)	4.4 (3.7–5.3)	
WTP		1,322 (36%)	2,374 (64%)		0.841	2,240	1,456		**<0.001**
	> 10,000 DKK	131 (10%)	248 (10%)	1 (ref)		330 (15%)	49 (3%)	1 (ref)	
	5,000–9,999 DKK	191 (14%)	328 (14%)	0.9 (0.7–1.2)		436 (20%)	83 (6%)	1.3 (0.9–1.9)	
	2,000–4,999 DKK	355 (27%)	681 (29%)	1.0 (0.8–1.3)		756 (34%)	280 (19%)	2.5 (1.8–3.5)	
	1,000–1,999 DKK	287 (22%)	494 (21%)	0.9 (0.7–1.2)		402 (18%)	379 (26%)	6.4 (4.6–8.9)	
	1–999 DKK	115 (9%)	199 (8%)	0.9 (0.8–1.3)		125 (6%)	189 (13%)	10.2 (7.1–15.0)	
	0 DKK	243 (18%)	424 (18%)	0.9 (0.7–1.2)		191 (9%)	476 (33%)	16.8 (12.0–23.9)	

		Space Availability				Resource Provision (gnawing opportunity)			
Owner variable	Category	*Above requirements n (%)*	*Below requirements n (%)*	Odds ratio (95% CI)	*P-value*	*Cont. opportunity n (%)*	*No cont. opportunity* *n (%)*	Odds ratio (95% CI)	*P-value*
Responsibility		187	398		0.354	3,641 (85%)	664 (15%)		**<0.001**
	Adult	120 (64%)	244 (61%)	1 (ref)		2,518 (69%)	428 (65%)	1 (ref)	
	All, equally	42 (23%)	110 (28%)	1.3 (0.9–2.0)		896 (25%)	154 (23%)	1.0 (0.8–1.2)	
	Child	25 (13%)	44 (11%)	0.9 (0.5–1.5)		227 (6%)	82 (12%)	2.1 (1.6–2.8)	
Starter Pet		184	395		0.856	3,601 (85%)	650 (15%)		**<0.001**
	Disagree	31 (17%)	69 (18%)	1 (ref)		1,399 (39%)	139 (21%)	1 (ref)	
	Partly disagree	35 (19%)	86 (22%)	1.1 (0.6–2.0)		693 (19%)	110 (17%)	1.6 (1.2–2.1)	
	Partly agree	61 (33%)	121 (31%)	0.9 (0.5–1.5)		915 (25%)	222 (34%)	2.4 (2.0–3.1)	
	Agree	57 (31%)	119 (30%)	0.9 (0.5–1.6)		594 (17%)	179 (28%)	3.0 (2.4–3.9)	
WTP		165	361		**0.019**	3,114 (84%)	582 (16%)		**<0.001**
	> 10,000 DKK					344 (11%)	35 (6%)	1 (ref)	
	5,000–9,999 DKK					469 (15%)	50 (8.6%)	1.1 (0.7–1.7)	
	2,000–4,999 DKK	43 (26%)	130 (36%)	1 (ref) (> 2,000) ^1^		920 (30%)	116 (20%)	1.2 (0.9–1.9)	
	1,000–1,999 DKK	51 (31%)	106 (29%)	0.7 (0.4–1.1)		646 (21%)	135 (23%)	2.1 (1.4–3.1)	
	1–999 DKK	27 (16%)	30 (8%)	0.4 (0.2–0.7)		251 (8%)	63 (11%)	2.5 (1.6–3.9)	
	0 DKK	44 (27%)	95 (26%)	0.7 (0.4–1.2)		484 (16%)	183 (31%)	3.7 (2.6–5.6)	

		Diet (hay/grass provision)				Veterinary Access			
Owner variable	Category	Ad libitum *hay* *n (%)*	*Not* ad libitum *hay* *n (%)*	Odds ratio (95% CI)	*P-value*	*Regular vet visits n (%)*	*Not regular vet visits n (%)*	Odds ratio (95% CI)	*P-value*
Responsibility		3,668 (85%)	637 (15%)			2,161	1,970		
	Adult	2,529 (69%)	417 (66%)	1 (ref)	**<0.001**	1,541 (71%)	1,297 (66%)	1 (ref)	**<0.001**
	All, equally	902 (25%)	148 (23%)	1.0 (0.8–1.2)		530 (25%)	474 (24%)	1.1 (0.9–1.2)	
	Child	237 (6.5%)	72 (11%)	1.8 (1.4–2.4)		90 (4%)	199 (10%)	2.6 (2.0–3.4)	
Starter Pet		3,622	629			2134	1949		
	Disagree	1,410 (39%)	128 (20%)	1 (ref)	**<0.001**	1,040 (49%)	477 (25%)	1 (ref)	**<0.001**
	Partly disagree	704 (19%)	99 (16%)	1.6 (1.2–2.0)		388 (18%)	386 (20%)	2.2 (1.8–2.6)	
	Partly agree	915 (25%)	222 (35%)	2.7 (2.1–3.4)		455 (21%)	618 (32%)	3.0 (2.5–3.5)	
	Agree	593 (16%)	180 (29%)	3.3(2.6–4.3)		251 (12%)	468 (24%)	4.1 (3.4–4.9)	
WTP		3147	549			1855	1,715		**<0.001**
	> 10,000 DKK	360 (11%)	19 (4%) ^2^	1 (ref)	**<0.001**	326 (18%)	48 (3%)	1 (ref)	
	5,000–9,999 DKK	486 (15%)	33 (6%)	1.3 (0.7–2.3)		386 (21%)	128 (8%)	2.3 (1.6–3.3)	
	2,000–4,999 DKK	922 (29%)	114 (21%)	2.3 (1.5–4.0)		635 (34%)	372 (22%)	4.0 (3.0–5.6)	
	1,000–1,999 DKK	647 (21%)	134 (24%)	3.9 (2.5–6.7)		311 (17%)	429 (25%)	9.4 (6.8–13.3)	
	1–999 DKK	242 (8%)	72 (13%)	5.6 (3.4–9.8)		82 (4%)	218 (13%)	18.1 (12.3–27.1)	
	0 DKK	490 (16%)	177 (32%)	6.8 (4.3–11.6)		115 (6%)	520 (30%)	30.7 (21.5–44.6)	

1 The categories ‘2,000–4,999’, ‘5,000–9,999’ and ‘> 10,000’ were merged into ‘> 2,000’ owing to few data points per level.

2 Kept as a category level albeit < 20 (confidence intervals not very broad).

P-values < 0.25 are highlighted in bold.

DKK: Danish crowns (1 DKK = 0.13 EUR; exchange rate per Jan. 2023).

Abbreviations: WTP, Willingness-To-Pay; CI, Confidence Interval.

###### Association of owner variables with husbandry conditions

Logistic regression was performed in R (R Core Team 2022) using the glm-function and the likelihood-ratio test for the assessment of statistical associations. Missing data and responses containing ‘uncertain’ or ‘other’ were excluded from the analysis. Pre-screening of variables was based on owner variables with *P <* 0.25 in univariable analysis. If the variable fulfilled this probability level, it was inserted into the multivariable model. Owner variables with *P* > 0.05 in the multivariable analysis were eliminated from the model, and from here owner variables with *P <* 0.05 in the final model were deemed significant. The Hosmer-Lemeshow goodness-of-fit test was applied to assess the fit of the final multivariable models. Confounding effects arising between the owner-variables (i.e. the independent variables) were determined as being present when there were ≥ 20% differences in the Odds Ratios (ORs) of the variables between the uni- and multivariable analyses.

#### Ethical considerations and approval (Surveys I and II)

Data from Survey I were pseudo-anonymised by Statistics Denmark (the researchers had no access to personal data). Respondents received information about the purpose of the project and consented to their responses being used for that purpose by clicking the survey link. In Survey II, the participant’s age and status as current owner of a rabbit was confirmed by preliminary survey questions. The dataset was anonymous and limited to include only data from respondents who completed the survey in full. Participants were told they could withdraw from the survey at any point by not completing the survey. Ethical approval was granted by the Research Ethics Committee of SCIENCE and HEALTH, University of Copenhagen (Survey I: case no 504-0246/21-5000, and Survey II: case no 504-0268/21-5000).

## Results

### Survey I

#### Owner information and rabbit population

A total of 2,347 (47%) respondents completed the survey. Based on the finding that 76 respondents reported keeping at least one rabbit, 2.9% (approximately 87,000) of Danish families were estimated to house an average (mean) of 1.9 rabbits, implying that approximately 163,000 (95% confidence interval; CI: 115,000–212,000) rabbits were being kept in private homes in Denmark in 2021. A majority of owners 83% (unweighted n = 60) reported the rabbit to be primarily considered as a companion animal, and 61% (unweighted n = 51) reported that the rabbit was acquired for a child/young adult (< 18 years).

#### Husbandry conditions

Of the 76 respondents, 23% kept the rabbit mainly indoors, 55% outdoors, 12% equally in- and outdoors, while 10% did not know how the rabbit was kept. Further, 71% of respondents housed the rabbit in a cage (16% used exclusive cage housing, 31% used cage housing with occasional access to another restricted area, 24% used cage housing with occasional free-roam access), 8% housed the rabbit in a restricted area such as a run (3% housed the rabbit exclusively in a restricted area, 5% in a restricted area with occasional free-roam access) and 9% housed the rabbit fully free to roam (9% did not know the answer to this question). Findings on owner-reported daily provision of rabbit resources included: social interaction with another rabbit (22%); digging opportunities (43%); gnawing opportunities (54%); places to hide (67%); rabbit toys (39%); and human contact or check-ups (72%). Further, 2% had provided none of the resources and 9% reported to not knowing whether they had done so. Neutering was reported for 22% of the rabbits, 11% were vaccinated each year, 22% received regular (annual) veterinary care, and none were health-insured.

### Survey II

#### Owner and rabbit information

A total of 4,335 respondents completed the survey for 4,335 companion rabbits (approximately 5% of Danish rabbit owners according to Survey I). Of these, 94.5% were female owners and 4.4% were male (0.5% chose ‘other’ and 0.6% preferred not to answer). The median age of the respondents was 38 years (interquartile range [IQR] 27–45). The most commonly reported types of residence were house/terraced house (51.0%), apartment (32.7%), and countryside residence/old farm (13.6%), and 58% of the respondents had children or young adults in their household (< 18 years) while 42% only had adults.

The median reported age of the 4,335 rabbits was two years (IQR 1–4) (with 1.5% of unspecified age). In all, 57.5% were male and 41.8% female (0.6% unknown). The median age of a previously owned rabbit at death was six years (IQR 4–8) (based on 2,609 responses). Further, 56.1% of the rabbits were neutered and 43% were intact (0.8% unknown) (by sex: 64.1% of males were neutered and 34.5% intact, while 46% females were neutered and 53.1% intact). The most common breed was dwarf lop (30.1%), followed by crossbred (13.6%), lionhead (11.2%), hermelin (4.9%), and 11.1% responded that they did not know the breed of their rabbit.

#### Husbandry conditions and outcome variables

Most rabbits were solitarily housed (64.9%; n = 2,809), with 32.5% (n = 1,408) being socially housed (i.e. with one or more conspecifics). One-third (33.3%; n = 509) of the socially housed rabbits were housed with intact partners, and one-third (32.1%; n = 1,309) of the rabbits were housed with other animals (i.e. sharing living space), mainly dogs (18.5%) and/or cats (15.1%). Approximately half (49.4%; n = 2,152) of the rabbits were mainly housed indoors and 39.4% (n = 1,707) were housed outdoors, while 11% (n = 476) were equally in- and outdoor housed (12.7% of the indoor rabbits were never let outside). Reported housing types were: 39.1% (n = 1,694) completely free-roam; 33.5% (n = 1,451) in a run/larger enclosure; (21% [n = 912] were kept exclusively in a run/larger enclosure and 12.4% [n = 539] given occasional free-roam access); 27.5% (n = 1,190) in a cage or hutch; 14.9% (n = 646) kept the rabbit in a cage with occasional access to a run/larger enclosure; 9.8% (n = 426) kept the rabbit in a cage with occasional access to free-roam; and 2.7% (n = 118) kept the rabbit in a cage at all times (Table S1). Approximately half of the rabbits housed in cages (53.4%; n = 635) had daily access to an area outside their cage for less than 7 h, and approximately half were reported to have no outside-cage access during active hours as defined for a crepuscular species (see Table S1 in Supplementary materials for detailed information on rabbit housing arrangements).

Detailed housing dimensions and stocking density relative to regulatory requirements laid down for laboratory rabbits (and recommendations by DB) are listed in Table 1. Of the respondents using cage housing and who entered information on cage dimensions, it was found that 9.1% (n = 34) housed their rabbits in cages or hutches below the threshold requirements for cage height, 67.7% (n = 399) housed their rabbits below the threshold for total area (m^2^) and 46.5% (n = 87) housed their rabbits below the threshold for stocking density (Table 1).

For 84.5% (n = 3,664) of the cases, rabbits were provided with continuous access to gnawing resources, while 15.5% (n = 671) had only occasional access or no such access. Further, 85.2% (n = 3,693) of the rabbits were provided with *ad libitum* hay or grass, 11.7% (n = 506) were provided with hay/grass daily but not *ad libitum*, and 3.1% (n = 136) were provided with hay/grass less often than daily. The most common feed concentrate was homogeneous pellets (provided daily: 70%). Muesli mix was provided daily by 11%. However, 23 and 6% of the respondents provided, respectively, pellets and muesli mix *ad libitum.* Among the owners, half (50.1%; n = 2,172) reported taking the rabbit to a veterinarian at least once a year (for detailed category results, see Table S1 in Supplementary materials). Most rabbits (80%) were not health-insured, with only 17.6% being covered by health insurance (2.4% unknown). Similarly, the majority of the rabbits had never been vaccinated (61.2%). Regular annual vaccination was reported for only 29.2% of rabbits, and less frequent vaccination was reported for 5% (4.6% were uncertain).

#### Reported owner reasons for social and spatial housing arrangements

Based on 1,526 responses, the commonest reported reasons (multiple-choice questions with multiple responses per respondent) for opting for solitary housing were: (1) not having the means or simply not wanting, to engage in the rabbit-bonding process (29.8%); (2) not having the resources (e.g. space, time or money) needed to keep several rabbits (25.7%); and (3) believing that rabbits thrive satisfactorily alone (17.4%). Based on 2,809 responses, the commonest owner-reasons for having social housing were: (1) to allow the rabbit to socialise (82.5%); (2) believing social housing to be essential for rabbits (71.0%); and (3) enjoying having several rabbits (18.2%). Based on 1,190 responses, the commonest owner-reasons for using cage housing were: (1) for the rabbit’s own safety (e.g. protection from electrical cords or predators) (61.0%); (2) enabling outdoor housing without the rabbit being able to escape (e.g. from the garden) (31.2%); and (3) managing the uncleanliness or destructive behaviour in the rabbit (17.2%). Where the adoption of semi-free and free-roam housing was concerned, the commonest owner-reasons, based on 3,145 responses, were: (1) believing rabbits need a lot of space to thrive (90.1%); (2) believing it benefits the human-animal relation to have the rabbit free-roaming (e.g. since then a positive rabbit-owner interaction is enabled) (54.4%); and (3) generally being against keeping animals in cages (52.8%). All owner-reported reasons for social housing arrangements can be found in Table S3 in the Supplementary materials.

#### Owner variables

Table S2 (Supplementary materials) contains detailed data on owner variables. Overall, 57.2% (n = 2,478) of rabbits were acquired for an adult (≥18 years) and 37.1% (n = 1,610) for children (<18 years), with the most common age group being 6–11 years. As regards the person in the household responsible for rabbit care, this was most often an adult (67.9%; n = 2,496) (predominantly the respondent: 64.6%; n = 2,801), followed by the responsibility being shared equally by all members of the household (24.2%; n =1,050). Approximately half (49.2%; n = 2,131) of the respondents reported WTP between 1 and 4,999 DKK and 41.1% (n = 1,796) reported WTP of 5,000 DKK and above. Just 15.4% (n = 667) reported WTP of nothing over 0 DKK (i.e. indicating that the respondent would forego surgery and ask for euthanasia). In response to the statement that rabbits are suitable as starter pets for children, 17.8% (n = 773) agreed, 26.2% (n = 1,137) partially agreed, 18.5% (n = 803) partly disagreed, and 35.5% (n = 1,538) disagreed (1.9%, n = 84 were uncertain) (NB 1DKK = 0.13€ as per January 2023).

#### Associations between owner variables and rabbit husbandry conditions

Univariable analyses of owner variables associated with rabbit husbandry conditions are shown in Table 2. Uni- and multivariable analyses resulted in five final models for the outcome variables Housing Type, Space Availability, Resource Provision, Diet and Veterinary Access (Table 3). None of the owner variables in the Social Housing model reached global significance. There were significant associations between Housing Type and all of the owner variables (*P <* 0.001). More specifically, the odds of cage/run housing were higher than the odds of partly/full free-roam housing when: (1) a child was responsible for rabbit care (‘Child’: *P <* 0.001); (2) the owner perceived rabbits as good starter pets (all categories: *P <* 0.001); and (3) when low[er] WTP was reported by the owner (all categories: *P <* 0.001, except ‘5,000–9,999 DKK’) (Table 3). Space Availability was significantly associated with WTP (*P =* 0.02), indicating lower odds of housing the rabbit in a cage below the threshold for spatial requirements when the reported WTP was ‘1–999 DKK’ rather than ‘> 2,000 DKK’ (*P =* 0.002) (Table 3). There were significant associations between Resource Provision and all the owner variables (Responsibility: *P <* 0.001; Starter Pet: *P <* 0.001; WTP: *P <* 0.001), indicating higher odds of not providing gnawing opportunities continuously when: (1) a child was responsible for rabbit care (‘Child’: *P <* 0.001); (2) the owner perceives rabbits as good starter pets (all categories: *P <* 0.001); and (3) when low(er) WTP was reported by the owner (‘0 DKK’ and 1–999 DKK’: *P <* 0.001) (Table 3). Significant associations were found between Diet and Starter Pet (*P <* 0.001) as well as WTP (*P <* 0.001). There were higher odds of not providing hay/grass *ad libitum* when: (1) the owner perceives rabbits as good starter pets (‘Partly Agree’: *P <* 0.001; ‘Agree’: *P* < 0.001); and (2) the WTP is low(er) (‘2,000–4,999 DKK’: *P =* 0.007; ‘1,000–1,999 DKK’: *P* < 0.001; ‘1–999 DKK’: *P* < 0.001; ‘0 DKK’: *P <* 0.001) (Table 3). Finally, there were significant associations between Veterinary Access and Starter Pet (*P <* 0.001) as well as WTP (*P <* 0.001). There were higher odds of not providing regular healthcare when: (1) the owner perceives rabbits as good starter pets (all categories *P <* 0.001); and (2) the WTP is low(er) (all categories: *P <* 0.001) (Table 3).

**Table 3.** Multivariable (and univariable) logistic regression analyses of the odds of rabbit husbandry not meeting recommendations relative to owner variables indicative of perceived low status of rabbits (n = 4,335)

*Outcomes (and included owner variables)*		Housing Type *~ Responsibility, Starter Pet, WTP*		Space Availability *~ WTP*		Resource Provision (gnawing opportunity) *~ Responsibility, Starter Pet, WTP*		Diet (hay/grass provision) *~ Starter Pet, WTP*		Veterinary Access *~ Starter Pet, WTP*	
Owner variable	Category	Odds ratio (95% CI)	*P-value*	Odds ratio (95% CI)	*P-value*	Odds ratio (95% CI)	*P-value*	Odds ratio (95% CI)	*P-value*	Odds ratio (95% CI)	*P*
	*Intercept*	0.1 (0.1–0.2)	**<0.001**	3.0 (2.2–4.3)	**<0.001**	0.1 (0.1–0.1)	**<0.001**	0.04 (0.03–0.1)	**<0.001**	0.1 (0.1–0.2)	**<0.001**
Responsibility	Adult	1 (ref)	**<0.001**	–	–	1 (ref)	**<0.001**	–	–	–	–
	All, equally	0.9 (0.8–1.1)	0.342	–	–	0.9 (0.7–1.1)	0.306	–	–	–	–
	Child	2.8 (2.1–3.8)*	**<0.001**	–	–	1.7 (1.2–2.3)*	**<0.001**	–	–	–	–
Starter Pet	Disagree	1 (ref)	**<0.001**	–	–	1 (ref)	**<0.001**	1 (ref)	**<0.001**	1 (ref)	**<0.001**
	Partly disagree	1.5 (1.2–1.9)*	**<0.001**	–	–	1.6 (1.2–2.1)	**<0.001**	1.3 (1.0–1.8)	**0.084**	1.6 (1.3–2.0)*	**<0.001**
	Partly agree	2.0 (1.7–2.5)*	**<0.001**	–	–	2.1 (1.6–2.7)	**<0.001**	2.0 (1.54–2.6)*	**<0.001**	1.9 (1.5–2.3)*	**<0.001**
	Agree	2.6 (2.1–3.3)*	**<0.001**	–	–	2.5 (1.9–3.3)	**<0.001**	2.5 (1.9–3.3)*	**<0.001**	2.4 (1.9–3.0)*	**<0.001**
WTP	≥ 10,000 DKK	1 (ref)	**<0.001**	1 (ref) (≥ 2000)^1^	**0.019**	1 (ref)	**<0.001**	1 (ref)	**<0.001**	1 (ref)	**<0.001**
	5,000-9,999 DKK	1.1 (0.8–1.7)	0.552			0.9 (0.6–1.5)	0.747	1.2 (0.7–2.2)	0.526	2.2 (1.5–3.2)	**<0.001**
	2,000-4,999 DKK	2.0 (1.4–2.8)*	**<0.001**			1.0 (0.7–1.5)*	0.854	1.2 (1.2–3.4)*	**0.007**	3.6 (2.6–5.1)	**<0.001**
	1,000-1,999 DKK	4.6 (3.3–6.7)*	**<0.001**	0.7 (0.4 –1.1)	0.126	1.4 (1.0–2.2)*	0.080	2.9 (1.8–5.0)*	**<0.001**	8.0 (5.7–11.5)	**<0.001**
	1-999 DKK	6.7 (4.6–9.9)*	**<0.001**	0.4 (0.2–0.7)	**0.002**	1.6 (1.0–2.5)*	**0.049**	4.0 (2.4–7.1)*	**<0.001**	14.0 (9.4–21.2)*	**<0.001**
	0 DKK	11.5 (8.1–16.5)*	**<0.001**	0.7 (0.4–1.2)	0.184	2.4 (1.6–3.6)*	**<0.001**	4.8 (3.0–8.2)*	**<0.001**	24.3 (16.8–35.8)*	**<0.001**

Housing Type: Levels ‘Free roam (full or partly)’ (reference level) and ‘Cage or run’; Space Availability: Levels ‘Above requirements’ (reference level) and ‘Below requirements’; Resource Provision: Levels ‘Continuous gnawing opportunity’ (reference level) and ‘No continuous gnawing opportunity’; Diet: Levels: ‘*Ad libitum* hay/grass’ (reference level) and ‘*Not ad libitum* hay/grass’; Veterinary Access: Levels ‘Regular visits’ (reference level) and ‘Not regular visits’.

Significant *P*-values are highlighted in bold (< 0.05).

1 The categories ‘2,000-4,999’, ‘5,000-9,999’ and ‘≥ 100,00’ were merged into ‘≥ 2,000’ owing to few data points per level.

* More than 20% difference in the OR of the variable between the uni- and multivariable analyses. DKK: Danish crowns (1 DKK = 0.13 EUR; exchange rate per Jan. 2023).

Abbreviations: WTP, Willingness-To-Pay; CI, Confidence Interval.

The Hosmer-Lemeshow goodness-of-fit test was non-significant for all models, indicating adequate model fits. Differences of ≥ 20% between the ORs of the uni- and multivariable indicating confounding effects were found for Responsibility, Starter Pet and WTP in the Housing Type model, for Responsibility and WTP in the Resource Provision model, for Starter Pet and WTP in the Diet model, and for Responsibility, Starter Pet and WTP in the Veterinary Access model (Table 3). Comparisons of model outputs revealed that Responsibility and WTP were confounded mainly by Starter Pet, and that Starter Pet was confounded mainly by WTP.

## Discussion

We investigated the husbandry conditions of companion rabbits relative to recommendations, as well as the effect of the owners’ perception of rabbits as low investment ‘children’s starter pets’ on the provision of such conditions, and hence on rabbit welfare. To the authors’ knowledge, this is the largest survey of companion rabbit welfare to date, and the first to combine these results with data from a representative survey. We found that a substantial proportion of rabbits are being housed in less than ideal conditions, such as solitarily and/or in inappropriately sized housing, and being provided with inadequate types of feed and other resources. Several of the conditions were associated with owners’ passing over responsibility for caring for the rabbit onto children, viewing the rabbit as a good ‘starter pet’ for them, and having low WTP for veterinary treatment. It has recently been claimed that companion rabbits are being afforded a higher status due to more adults acquiring them, and because they are now more highly valued by their owners (e.g. at the veterinary clinic; PDSA 2020) and are increasingly being provided with free-roam housing (e.g. Buseth & Saunders 2015; Mayer *et al.* 2017; McMahon & Wigham 2020). However, our results indicate that most rabbits, at least in Denmark, are still considered ‘children’s pets’, do not receive preventive healthcare and are cage-housed.

### Conditions for companion rabbits and highlighted welfare concerns

We found rabbits to be among the most popular companion animals in Denmark, present in approximately 3% of households. This finding is in accordance with previous studies from Northern Europe (Schepers *et al.* 2009; Mäkitaipale *et al.* 2015; Ulfsdotter *et al.* 2016; PFMA 2019; Mee *et al.* 2022; PDSA 2022). The average lifespan of a companion rabbit was found to be six years, in line with reports from the UK of 5.6 years (Rooney *et al.* 2014) and the Netherlands of 6.7 years (Vink *et al.* 2018). Despite probably being an overestimation (based on a convenience sample of Survey II which overrepresented high standard rabbit care and housing – to be discussed further below), this is a relatively short lifespan compared to the potential of 8–14 years (depending on breed characteristics) (Altman & Dittmer 1972; Schepers *et al.* 2009; Buseth & Saunders 2015; Ulfsdotter *et al.* 2017). Factors contributing to the reduced number of years may include low investment in healthcare and poor husbandry. The latter may include solitary housing (Schepers *et al.* 2009) and non-recommended feeding regimes such as limited access to hay/grass, feeding muesli mix and providing pellets *ad libitum* (e.g. Rioja-Lang *et al.* 2019). All of these factors emerged as prevalent in this study. We found approximately two-thirds of rabbits being housed solitarily in Survey II. The proportion was even higher in the representative Survey I (78%) and significantly exceeded the already high numbers reported in nearby countries: for example, 45% in the UK and the Netherlands (Mullan & Main 2006; Schepers *et al.* 2009). Solitary housing has been identified as a serious welfare problem for companion rabbits (Rioja-Lang *et al.* 2019). Rabbits kept with conspecifics display less stereotypy and fear than those kept solitarily (Mullan & Main 2006, 2007; Schepers *et al.* 2009; Burn & Shields 2020). Social housing is a requirement for rabbits used in experimental research and in food production (DiVincenti & Rehrig 2016; Danish Ministerial Order regarding Animal Experimentation 2021). Despite the well-documented welfare challenges presented by solitary housing, approximately one in five of the owners in Survey II reported that they housed their rabbits without a partner since they believed rabbits thrived satisfactorily alone.

A large share of the rabbits in Survey II were reported to be housed as completely free-roam rabbits, indicating alternative approaches to rabbit housing, compared to the more traditional cage housing. However, results from the representative Survey I showed cage housing to still be the dominant arrangement, and we found that a large proportion of the respondents had housed their rabbits in cages below minimum requirements and/or recommendations on dimensions (Survey II). Restricted space hampers behavioural expression and increases stereotypic behaviour, compromising rabbit welfare (Dixon *et al.* 2010; Normando & Gelli 2011). By contrast, increased space leads to more activity, foraging, environmental interaction and play (Mullan & Main 2007; Dixon *et al.* 2010). The proportion of cages we saw with dimensions below those laid down in requirements/recommendations indicates that rabbit cages and hutches currently on the market are not conducive to rabbit activity and may even be unsuitable for rabbits in other sectors. Given our findings that half of these rabbits do not have outside-cage access during active hours between dawn and dusk (Díez *et al.* 2013), and that almost half have outside-cage access for less than 7 h daily – something that may increase stereotypic behaviour (Normando & Gelli 2011) – unsuitable caging probably compromises the welfare of many rabbits.

Since the most common reasons for opting for cage housing relate to the belief that this type of housing enables the owner to physically restrict the rabbit for its own safety, it is plausible that owners would be less likely to physically restrict their rabbits if they were given information on alternative housing solutions. However, at the same time, the reported owner-reasons highlight a relatively low willingness among owners to invest in rabbit housing (e.g. by not using protectors for electrical cords indoors or fencing to allow outside-cage roaming) (Table S3; Supplementary materials). Limited willingness to invest was also a common theme among the reasons reported for not providing social housing, as additional resources were expected by the owners to be required to be able to house multiple rabbits or for bonding rabbits. The owners’ willingness to invest in rabbit husbandry may therefore be an important factor affecting rabbit welfare, as the results of our regression analyses also suggest.

### Perceived status of rabbits and its effect on provision of husbandry conditions

Our Survey I results show that most rabbits are still acquired for children. Given the results from Survey II, indicating that nearly half (44%) of the respondents agreed (partly or fully) that rabbits are appropriate ‘starter pets’ for children, it is clear that many owners still consider rabbits a ‘low status’ companion animal that requires a low level of care. Being considered a ‘children’s pet’ has been reported to put rabbits at risk of receiving little care (Rioja-Lang *et al.* 2019; PDSA 2020), and our results from Survey I indicate that nearly one-third of rabbits are not being checked daily or socialised by a human, indicating potential neglect of many rabbits.

In line with our hypothesis, we found that owners who consider rabbits to be of low status are less likely to provide appropriate conditions relative to recommendations. The results showed that owners who perceive rabbits as ‘starter pets’ and have low WTP are more likely to house their rabbits in restricted space (housing type), and to fail to provide continuous gnawing opportunities, *ad libitum* hay, and/or routine healthcare. A child being responsible for rabbit care had a negative effect solely on housing type and gnawing opportunities. The impact of perceived rabbit status on the resources being provided was also highlighted in the models by increasing odds (ORs) per category level that increasingly reflected lower rabbit status (an exception here was Space Availability, which may be an accidental finding, and specifically a Type-1 error, possibly brought about by self-selection bias). No such relationship was found between social housing and the owner variables, suggesting that solitary housing may be independent of an owner’s ascription of low status to rabbits. Since many owners reported housing their rabbit alone because they believed that the rabbit was enriched by other husbandry conditions (e.g. free-roam access), the importance of social housing for rabbits should be disseminated to all owner types.

In contrast with most other companion animal species, rabbits are used for various purposes in society (e.g. experimental research, hobby breeding and showing, as feed for other species, in the training of hunting dogs, and in farming) and are considered a pest in some countries. The fact that rabbits have multiple roles in society probably lowers their rank on the socio-zoological scale. This may reduce the overall human attribution of rabbits’ mental abilities as well as the perceived moral obligation towards rabbits (Loughnan *et al.* 2010). Ultimately, it may have negative impacts on the human investment in rabbit care. This risk has been highlighted in previous studies (Edgar & Mullan 2011; McMahon & Wigham 2020), which found that the owners’ perception that rabbits have limited mental abilities compromises rabbit husbandry and welfare. Failure to provide an environment that enables and promotes natural rabbit behaviour, and which instead promotes apathy and boredom (e.g. imposed by barren-cage housing), may wrongly confirm, and even accentuate, the perception among owners of rabbits having limited mental abilities and simple needs.

This study asked rabbit owners the extent to which they agreed with rabbits being good ‘starter pets’ and ‘childrens’ pets.’ Although many rabbits were cared for primarily by an adult, there was a rather large variation in owner opinions on this topic. It would be beneficial if future research investigates the meaning of these terms to owners, including which characteristics of a species that contribute to it being coined as a ‘starter’ or ‘childrens’ pet’ (and what characteristics are associated with the opposite, i.e. demanding and not suitable for children). Such information could help drive improvements in general advice given on rabbit (and other small companion animal) care and provide realistic recommendations on rabbit husbandry.

### Study limitations

The data from Survey II were collected through Facebook, introducing self-section bias towards a certain type of owner: for example, rabbit-enthusiastic and dedicated owners with access and time to be active on the internet (McMahon & Wigham 2020). By using the results from Survey I as a benchmark it was possible to assess the sample misrepresentation in Survey II. This comparison indicated a substantial overrepresentation, in Survey II, of owners who kept their rabbit indoors (22.5% in Survey I vs 49.6% in Survey II) and as free-roaming (9 vs 39%). This misrepresentation, involving potentially more dedicated rabbit owners (housing free-roaming rabbits requires additional resources in terms of space provision, time, and rabbit-proofing, among other things), probably led to an overestimation, in Survey II, of the proportion of owners who keep their rabbits in appropriate conditions. There were clearly more female (95%) than male (5%) responders in Survey II, as has been reported in previous rabbit surveys: for example, 94% female respondents in McMahon and Wigham (2020) and 89% female respondents in Rooney *et al.* (2014). This could be either because rabbits are more likely to be kept by female owners (Mee *et al.* 2022) or because females may be more prone to participate in surveys (Smith 2008). We can point out the actual source of the gender distribution by comparing Survey II with the patterns in Survey 1. Here, there were approximately the same proportion of men (3.3%) as women (3.6%) that reported to have a rabbit in the family. This is a further indication that the Survey II sample suffers from strong (self) selection bias. *Post hoc* analysis of the data from Survey I revealed that women were more likely than men to report being the person in the household: (1) most eager to acquire the rabbit (10% women vs 2% men; other responses included 46% children, 15% everyone in the household and 27% other); (2) spending the most time on activities with the rabbit (17% women vs 5% men); (3) feeding the rabbit the most (18% women vs 5% men); and (4) most attached to the rabbit (13% women vs 4% men). This may explain part of the pronounced bias towards female participation that is often reported in rabbit surveys. Nonetheless, the overrepresentation of females may clearly limit the generalisability of the results, since female rabbit owners are more likely to provide adequate rabbit housing than males (Mee *et al.* 2022), further leading to an underestimation of the share of rabbits living in unsuitable conditions in Survey II. Such an underestimation could, in turn, have obscured the identification of significant associations between husbandry conditions and owner variables. Moreover, previous studies have found evidence of social desirability bias in rabbit owners, established through misalignments with on-site visits and survey data, resulting in an overestimation of, among other things, social housing and appropriate feeding regimes (Mullan & Main 2006; Vink *et al.* 2018). Appropriate conditions may therefore have been overestimated in both of these surveys here. The fact that categories were merged for both outcomes and owner-related variables because there few data-points per category may also have impacted upon our findings by reducing the degree of detail contained at each category level. However, in general, because we tested our hypothesis against several aspects of rabbit husbandry (social housing, housing type, space availability, resource provision and veterinary access), and because we found that owners who perceived the rabbit’s status as low were more likely to provide their animals with inadequate conditions for several of these aspects, the risk of spurious results may have been reduced.

Although the general sample size was large, some variables (e.g. spatial dimensions) had fewer data-points than others, reducing the reliability of the corresponding results. The accuracy of the spatial variables is also likely to have been compromised, as they are self-reported by owners. Correct measurements and units may not have been entered in all cases, possibly resulting in an overestimation of spatial dimensions. Finally, it is important to note that some respondents may not have been the main providers of care for the rabbits, and consequently may not have entered the correct answers to certain questions. There was a relatively high occurrence of the option ‘Don’t know’ across many of the questions in Survey I. We assume that this stems from a random person within the household being recruited to partake in the study. Some of these respondents will therefore not be the primary caretaker and may undertake very little engagement with the rabbit or even be unaware of the actual conditions.

### Animal welfare implications

In this study, a large proportion of companion rabbits were found to be kept in inadequate conditions potentially compromising their welfare. The conditions include solitary housing, failure to provide appropriate rabbit resources, space availability below recommended levels, failure to provide routine healthcare and indications of daily neglect. A driving factor behind these conditions is the owners’ perception that the rabbit is a low investment ‘starter pet’: we confirmed that such perceptions increase the risk of inappropriate rabbit husbandry. The results presented here show how the status of a companion animal, as perceived by the owner, can impact animal welfare. The results thus emphasise the need to target human perceptions of rabbits in efforts to raise standards of rabbit welfare. The existing owner perceptions of rabbits may be advantageously altered by ceasing the common depiction of rabbits alongside small children and in small cages, which may misleadingly signal the rabbit as a low effort and low-cost companion animal. Instead, showcasing and encouraging natural, complex and diverse rabbit behaviour that can be seen when they are offered adequate space and enrichment (McMahon & Wigham 2020), may positively improve owner perceptions and, in turn, rabbit husbandry. It is also clear that the lack of legal protection and official recommendations on rabbit care renders rabbits vulnerable to inappropriate husbandry. This study underscored this by showing the large proportion of rabbits currently being kept in inadequate conditions. The promotion of official codes of practice, and guidelines stressing the required level of investment for appropriate rabbit care, may further help to improve rabbit welfare.

## Supplementary material

For supplementary material accompanying this paper visit https://doi.org/10.1017/wf.2023.41. click here to view supplementary material
