# Supplementary material for: Perceptions of the rabbit as a low investment ‘starter pet’ lead to negative impacts on its welfare: Results of two Danish surveys
Source: Anim Welf. 2023 Jul 3;32:e45. doi: 10.1017/awf.2023.41 (PMC10936283; doi:10.1017/awf.2023.41)
Supplement: Supplementary file 1 [file S0962728623000416sup001.docx]

**Content of supplementary materials**

Table S1 **2**

Table S2 **5**

Table S3 **6**

Table S4 **8**

English version of the questionnaire for Survey I **10**

English version of the questionnaire for Survey II **13**

| ***Topic*** | *n* | % | |
| --- | --- | --- | --- |
| **Social housing with other rabbit(s) vs. solitary housing** | *4,335* | |  |
| Social housing | (1,526) | | 35.5 |
| *Social housing - continuously* | *1408* | | *32.5* |
| *Social housing - occasionally, daily* | *118* | | *2.7* |
| Solitary housing | 2,809 | | 64.9 |
| *Solitary housing - possibility of tactile contact* | *220* | | *5.1* |
| *Solitary housing - possibility of visual contact* | *159* | | *3.7* |
| *Solitary housing - rabbit is kept alone* | *2,430* | | *56.1* |
|  |  | |  |
| **Neuter status of partner(s)** | *1,526* | |  |
| Partner rabbit(s) neutered | 1,006 | | 65.9 |
| Only some partner rabbit(s) neutered | 48 | | 3.1 |
| Partner rabbit(s) unneutered | 461 | | 30.2 |
| Unknown | 11 | | 0.7 |
|  |  | |  |
| **Housing with other species** ^(multiple choice)^ | *4,335* | |  |
| Housed with other species: | 1,390 | | 32.1 |
| *Dog* | *801* | | *18.5* |
| *Cat* | *654* | | *15.1* |
| *Rodent* | *106* | | *2.4* |
| *Chicken* | *99* | | *2.3* |
| *Other bird* | *84* | | *1.9* |
| *Reptile or amphibian* | *26* | | *0.6* |
| *Other animal* | *57* | | *1.3* |
| Not housed with other species | 2945 | | 67.9 |
| **Housing (restricted or free roam)*** | *4,335* | |  |
| Cage-housed ^a^ | 118 | | 2.7 |
| Cage-housed w. restricted area access ^b^ | 646 | | 14.9 |
| Caged-housed w. free-roam access ^c^ | 426 | | 9.8 |
| Run/enclosure-housed ^d^ | 912 | | 21 |
| Run/enclosure-housed w. free-roam access ^e^ | 539 | | 12.4 |
| Free roam ^f^ | 1,694 | | 39.1 |
|  |  | |  |
| **Indoor/outdoor housing** | *4,335* | |  |
| Indoor | 2,152 | | 49.6 |
| *Exclusively indoor* | *1,323* | | *30.5* |
| *Primarily indoor* | *829* | | *19.1* |
| Outdoor | 1,707 | | 39.4 |
| *Exclusively outdoor* | *1,380* | | *31.8* |
| *Primarily outdoor* | *327* | | *7.5* |
| Equally indoor and outdoor | 476 | | 11 |
|  |  | |  |

**Table S1**. Frequency and percentages for companion rabbit husbandry parameters included in the study (prior re-coding of outcome categories) (Survey II).

Multiple choice: Respondents could choose multiple response options (i.e. frequencies do no add to 100%)

| ***Topic*** | *n* | % |
| --- | --- | --- |
| **Outdoor-access frequency** (indoor rabbits) | *2,628* |  |
| Continuously/several times a day | 390 | 14.8 |
| Daily | 587 | 22.3 |
| Several times a week | 399 | 15.2 |
| Weekly | 254 | 9.7 |
| Monthly | 208 | 7.9 |
| Less than monthly | 455 | 17.3 |
| Never | 335 | 12.7 |
|  |  |  |
| **Daily time outside cage** | *1,190* |  |
| Does not typically come out of the cage daily | 67 | 5.6 |
| Under 1 hour | 73 | 6.1 |
| 1 - 3 hours | 261 | 21.9 |
| 4 - 6 hours | 234 | 19.7 |
| 7 - 9 hours | 168 | 14.1 |
| 10 - 12 hours | 115 | 9.7 |
| Over 12 hours | 220 | 18.5 |
| Uncertain | 52 | 4.4 |
|  |  |  |
| **Time of day outside cage** ^(multiple choice)^ | *1,190* |  |
| Morning: |  |  |
| Not out | 675 | 56.7 |
| Out | 515 | 43.3 |
| Late morning: |  |  |
| Not out | 731 | 61.4 |
| Out | 459 | 38.6 |
| Afternoon: |  |  |
| Not out | 242 | 20.3 |
| Out | 948 | 79.7 |
| Evening: |  |  |
| Not out | 507 | 42.6 |
| Out | 683 | 57.4 |
| Night: |  |  |
| Not out | 1,087 | 91.3 |
| Out | 103 | 8.7 |
| Uncertain | 46 | 3.9 |

**Table S1**. (continued)

*The respondents were informed that rabbits with permanently open cages should not count as cage-housed a Cage-housed without access to other area (e.g. run/house); b Cage-housed with access to a restricted area access (e.g. run/other enclosure) at certain times; c Cage-housed with access to the whole/most of the home or garden at certain times; d Housed in a restricted area (e.g. run/other enclosure) all the time; e Housed in a restricted area (e.g. run/other enclosure) with access to the whole/most of the home or garden at certain times
f Free roam in the whole/most of the home or garden all the time
Multiple choice: Respondents could chose multiple response options (i.e. frequencies do no add to 100%)

**Table S1**. (continued)

| ***Topic*** | ***n*** | ***%*** |
| --- | --- | --- |
| **Hay/grass provision** | *4,335* |  |
| Ad libitum/available all the time | 3,693 | 85.2 |
| Daily | 506 | 11.7 |
| Several times a week | 95 | 2.2 |
| Weekly | 25 | 0.6 |
| Monthly | 10 | 0.2 |
| Less than monthly | 3 | 0.1 |
| Never | 3 | 0.1 |
|  |  |  |
| **Provision of opportunity to gnaw** | *4,335* |  |
| Continuously | 3,664 | 84.5 |
| Occasionally | 632 | 14.6 |
| Never | 39 | 0.9 |
|  |  |  |
| **Veterinary visits** | *4,335* |  |
| Twice a year | 877 | 20.2 |
| Once a year | 1295 | 29.9 |
| Every second year | 115 | 2.7 |
| Less than every second year | 147 | 3.4 |
| Only in case of acute illness/disease | 1670 | 38.5 |
| Never, even in case of acute illness/disease | 56 | 1.3 |
| Uncertain | 175 | 4.0 |

Multiple choice: Respondents could choose multiple response options (i.e. frequencies do no add to 100%)

**Table S2**. Frequency and percentages of the owner-related variables, including type of person in the household the rabbit was acquired for, the person responsible for the rabbit, owner-reported opinion on rabbit suitability and Willingness-To-Pay (prior recoding) (Survey II).

| ***Topic*** | *n* | % |
| --- | --- | --- |
|  |  |  |
| **Person the rabbit is acquired for:** ^1^ | *4,335* |  |
| Myself/other adult (> 18 years) | 2,478 | 57.2 |
| Child 0-5 years | 164 | 3.8 |
| Child 6-11 years | 1,021 | 23.6 |
| Child/teenager 12-17 years | 425 | 9.8 |
| Other | 247 | 5.7 |
|  |  |  |
| **Person responsible for the rabbit:** | *4,335* |  |
| Myself (> 18 years) | 2,801 | 64.6 |
| Cohabitant/other adult (> 18 years) | 145 | 3.3 |
| Child 6-11 years | 125 | 2.9 |
| Child/teenager 12-17 years | 184 | 4.2 |
| Equally distributed in the family | 1,050 | 24.2 |
| Other | 30 | 0.7 |
|  |  |  |
| **Suitability as a starter pet for children** | *4,335* |  |
| Agree | 773 | 17.8 |
| Partly agree | 1,137 | 26.2 |
| Partly disagree | 803 | 18.5 |
| Disagree | 1,538 | 35.5 |
| Uncertain | 84 | 1.9 |
|  |  |  |
| **Willingness-To-Pay (WTP)** ^2^ | *4,335* |  |
| I would not pay and ask for euthanasia | 667 | 15.4 |
| Up to1999 DKK | 1,095 | 25.3 |
| 2000-4999 DKK | 1,036 | 23.9 |
| 5000-9999 DKK | 1,038 | 23.9 |
| 10000 DKK or more | 758 | 17.5 |
| Uncertain | 639 | 14.7 |

1 Owing to formulation of question as “Who in the household is the rabbit procured for?”, the age may not reflect the child’s age at acquisition.
2 Willingness-to-Pay at the veterinarian for life-saving rabbit surgery for both health insured and uninsured rabbits; responses for health insured (*n*=763) and not health insured rabbits *(n*=3,572) were merged.

**Table S3**. Frequency and percentages for owner reported reasons for rabbit housing arrangements (Survey II).

| ***Topic*** | *n* | % | |
| --- | --- | --- | --- |
|  |  | |  |
| **Reason(s) for social housing** ^(multiple choice)^ | *1,526* | |  |
| To enable the rabbits to socialise | 1,259 | | 82.5 |
| Rabbits should generally be/thrive better with other rabbits | 1,083 | | 71.0 |
| Because I like rabbits and to have multiple of them | 277 | | 18.2 |
| Because I found an additional rabbit that I wanted to have/provide a home for | 184 | | 12.1 |
| So that I do not have to interact as much with the rabbits (they entertain each other) | 210 | | 13.8 |
| So that I can breed them | 15 | | 1.0 |
| The rabbits are related/were already bonded when I acquired them ^N^ | 18 | | 1.2 |
| The children in the household have one rabbit each ^N^ | 10 | | 0.7 |
| Uncertain | 2 | | 0.1 |
| Other | *10* | | 0.7 |
|  |  | |  |
| **Reason(s) for solitary housing/keeping a single rabbit** ^(multiple choice)^ | *2,809* | |  |
| Rabbits should be/thrive better alone | 133 | | 4.7 |
| The rabbit has become alone (e.g. death of partner) and I do not want more rabbits | 292 | | 10.4 |
| It is difficult/not possible to house the rabbit with a partner (e.g. behavioural/health issues) | 486 | | 17.3 |
| Because I do not want to neuter the rabbit/rabbits | 285 | | 10.1 |
| Rabbits thrive decently alone | 488 | | 17.4 |
| It requires more resources (e.g. space/time/money) to have more rabbits | 721 | | 25.7 |
| I do not have the means to bond rabbits (e.g. spatial limitations), do not want to, or am too  nervous to bond rabbits | 837 | | 29.8 |
| I am nervous that my rabbit will become less social/tame | 131 | | 4.7 |
| The rabbit is temporarily alone and/or I am currently looking for another rabbit, for the rabbit | 272 | | 9.7 |
| I was misinformed about (appropriate) social housing of rabbits at acquisition ^N^ | 16 | | 0.6 |
| The rabbit is housed with another species ^N^ | 74 | | 2.6 |
| Because the rabbit is a free roaming rabbit, it is enriched from us humans or via other means ^N^ | 35 | | 1.2 |
| I only want one rabbit ^N^ | 22 | | 0.8 |
| The rabbit has always been housed alone ^N^ | 16 | | 0.6 |
| I was told by the seller/breeder/when purchasing the rabbit, that the rabbit should be housed  alone ^N^ | 17 | | 0.6 |
| Uncertain | 82 | | 2.9 |
| Other | 82 | | 2.9 |

Multiple choice: Respondents could choose multiple response options (i.e. frequencies do no add to 100%)
N: Novel category made over open-ended responses.
The frequencies and percentages reported for the categories “Uncertain” and “Other” do not include the response where another existing option was chosen by the respondent (or later by the rater during recoding).

| ***Topic*** | *n* | % | |
| --- | --- | --- | --- |
| **Reasons(s) for cage housing** ^(multiple choice)^ | *1,190* | |  |
| Rabbits do not need a lot of space | 6 | | 0.5 |
| The rabbit is not clean (house soiling), is loud or destroy things (e.g. furniture) | 205 | | 17.2 |
| For the rabbit's own safety (e.g. electrical cords or predators) | 726 | | 61.0 |
| The rabbit is aggressive or fearful (e.g. towards people or animals) | 24 | | 2.0 |
| To avoid unwanted mating | 42 | | 3.5 |
| To keep the rabbit clean (e.g. angora rabbit) | 7 | | 0.6 |
| So that the rabbit can come outdoors without running away | 371 | | 31.2 |
| Other | 121 | | 10.2 |
| Uncertain | 35 | | 2.9 |
|  |  | |  |
| **Reason(s) for free roam access** ^(multiple choice)^ | *3,145* | |  |
| Rabbits need a lot of space to thrive | 2,835 | | 90.1 |
| Rabbit cages takes up too much space/are not aesthetical pleasing | 73 | | 2.3 |
| I am generally against keeping animals in cages | 1,660 | | 52.8 |
| I get more out of my rabbit when free roaming (e.g. social interaction) | 1,710 | | 54.4 |
| The rabbit used to be cage housed but did not thrive (e.g. bar biting or cage-aggressiveness) | 165 | | 5.2 |
| Uncertain | 20 | | 0.6 |
| Other | 27 | | 0.9 |

**Table S3**. (continued)
Multiple choice: Respondents could choose multiple response options (i.e. frequencies do no add to 100%)
N: Novel category made over open-ended responses.
The frequencies and percentages reported for the categories “Uncertain” and “Other” do not include the response where another existing option was chosen by the respondent (or later by the rater during recoding).

**Table S4.** Descriptive data per variable category of the outcomes and the owner-related variables (prior recoding of owner-related variable categories).

| ***Owner variable*** | ***Category*** | ***Social Housing*** | | ***Housing Type*** | | ***Space Availability (m^2^)*** | | ***Resource Provision  (gnawing opportunity)*** | |
| --- | --- | --- | --- | --- | --- | --- | --- | --- | --- |
|  |  | ***Social***  *n = 1526* | ***Solitary***  *n = 2809* | ***Free roam (full or partly)***  *n = 2659* | ***Cage or Run***  *n = 1676* | ***Above Requirements***  *n =* *190* | ***Below Requirements***  *n = 399* | ***Continuous***    *n =* *3664* | ***Not Continuous*** *n = 671* |
| **Responsibility** | Adult, respondent  (> 18 years) | 1004 (65.8%) | 1797 (64.0%) | 1807 (68%) | 994 (59.3%) | 111 (58.4%) | 230 (57.6%) | 2403 (65.6%) | 398 (59.3%) |
|  | Adult, cohabitant/other (> 18 years) | 45 (2.9%) | 100 (3.6%) | 76 (2.9%) | 69 (4.1%) | 9 (4.7%) | 14 (3.5%) | 115 (3.1%) | 30 (4.5%) |
|  | Child (12-17 years) | 57 (3.7%) | 127 (4.5%) | 66 (2.5%) | 118 (7%) | 17 (8.9%) | 28 (7%) | 135 (3.7%) | 49 (7.3%) |
|  | Child (6-11 years) | 42 (2.8%) | 83 (3.0%) | 25 (0.9%) | 100 (6%) | 8 (4.2%) | 16 (4%) | 92 (2.5%) | 33 (4.9%) |
|  | Child (0-5 years) | 0 (0.0%) | 0 (0.0%) | 0 (0.0%) | 0 (0.0%) | 0 (0.0%) | 0 (0.0%) | 0 (0.0%) | 0 (0.0%) |
|  | Equally distributed in the household | 373 (24.4%) | 667 (24.1%) | 669 (25.2%) | 381 (22.7%) | 42 (22.1%) | 110 (27.6%) | 896 (24.5%) | 154 (23%) |
|  | Other | 25 (0.9%) | 5 (0.3%) | 16 (0.6%) | 14 (0.8%) | 3 (1.6%) | 1 (0.3%) | 23 (0.6%) | 7 (1.0%) |
| **Starter Pet** | Agree | 250 (16.4%) | 523 (18.6%) | 332 (12.5%) | 441 (26.3%) | 57 (30.0%) | 119 (29.8%) | 594 (16.2%) | 179 (26.7%) |
|  | Partly agree | 406 (26.6%) | 731 (26%) | 586 (22%) | 551 (32.9%) | 61 (32.1%) | 121 (30.3%) | 915 (25.0%) | 222 (33.1%) |
|  | Partly disagree | 273 (17.9%) | 530 (18.9%%) | 510 (19.2%) | 293 (17.5%) | 35 (18.4%) | 86 (21.6%) | 693 (18.9%) | 110 (16.4%) |
|  | Disagree | 574 (37.6%) | 964 (34.3%) | 1183 (44.5%) | 355 (21.2%) | 31 (16.3%) | 69 (17.3%) | 1399 (38.2%) | 139 (20.7%) |
|  | Uncertain | 23 (1.5%) | 61 (2.2%) | 48 (1.8%) | 36 (2.1%) | 6 (3.2%) | 4 (1.0%) | 63 (1.7%) | 21 (3.1%) |
| **WTP** | 0 (would ask for euthanasia) | 243 (15.9%) | 424 (15.1%) | 191 (7.2%) | 476 (28.4%) | 44 (23.2%) | 95 (23.8%) | 484 (13.2%) | 183 (27.3%) |
|  | 1-999 DKK | 115 (7.5%) | 199 (7.1%) | 125 (4.7%) | 189 (11.3%) | 27 (14.2%) | 30 (7.5%) | 251 (6.9%) | 63 (9.4%) |
|  | 1000-1999 DKK | 287 (18.8%) | 494 (17.6%) | 402 (15.1%) | 379 (22.6%) | 51 (26.8%) | 106 (26.6%) | 646 (17.6%) | 135 (20.1%) |
|  | 2000-4999 DKK | 355 (23.3%) | 681 (24.2%) | 756 (28.4%) | 280 (16.7%) | 28 (14.7%) | 80 (20.1%) | 920 (25.1%) | 116 (17.3%) |
|  | 5000-9999 DKK | 191 (12.5%) | 328 (11.7%) | 436 (16.4%) | 83 (5%) | 13 (6.8%) | 27 (6.8%) | 469 (12.8%) | 50 (7.5%) |
|  | 10000-29999 DKK | 65 (4.3%) | 129 (4.6%) | 170 (6.4%) | 24 (1.4%) | 1 (0.5%) | 13 (3.3%) | 170 (4.6%) | 24 (3.6%) |
|  | 30000-59999 DKK | 20 (1.3%) | 23 (0.8%) | 39 (1.5%) | 4 (0.2%) | 0 (0.0%) | 4 (1.0%) | 40 (1.1%) | 3 (0.4%) |
|  | 60000-99999 DKK | 10 (0.7%) | 18 (0.6%) | 22 (0.8%) | 6 (0.4%) | 0 (0.0%) | 2 (0.5%) | 26 (0.7%) | 2 (0.3%) |
|  | > 100000 DKK | 36 (2.4%) | 78 (2.8%) | 99 (3.7%) | 15 (0.9%) | 1 (0.5%) | 4 (1.0%) | 108 (2.9%) | 6 (0.9%) |
|  | Uncertain | 204 (13.4%) | 435 (15.5%) | 419 (15.8%) | 220 (13.1%) | 25 (13.2%) | 38 (9.5) | 550 (15.0%) | 89 (13.3%) |

Responsibility: The main person responsible for the care of the rabbit in the household; Starter Pet: Owner-opinion on the suitability of rabbits as first time companion animals for children; WTP: Willingness-To-Pay for life-saving veterinary treatment.
DKK: Danish crowns (1 DKK = 0.13 EUR; exchange rate per Jan. 2023).

| ***Owner variable*** | ***Category*** | ***Diet  (hay/grass provision)*** | | ***Veterinary Access*** | |
| --- | --- | --- | --- | --- | --- |
|  |  | ***Ad libitum hay/grass***  *n = 3693* | ***Not*** ***Ad libitum hay/grass*** *n = 642* | ***Regular visits***  *n = 2172* | ***Not*** ***Regular visits***  *n = 1988* |
| **Responsibility** | Adult, respondent (> 18 years) | 2411 (65.3%) | 390 (60.7) | 1484 (68.3%) | 1213 (61%) |
|  | Adult, cohabitant/other (> 18 years) | 118 (3.2%) | 27 (4.2%) | 57 (2.6%) | 84 (4.2%) |
|  | Child (12-17 years) | 148 (4.0 %) | 36 (5.6%) | 63 (2.9%) | 110 (5.5%) |
|  | Child (6-11 years) | 89 (2.4%) | 36 (5.6%) | 27 (1.2%) | 89 (4.5%) |
|  | Child (0-5 years) | 0 (0.0%) | 0 (0.0%) | 0 (0.0%) | 0 (0.0%) |
|  | Equally distributed in the household | 902 (24.4%) | 148 (23.1%) | 530 (24.4%) | 474 (23.8%) |
|  | Other | 25 (0.7%) | 5 (0.8%) | 11 (0.5%) | 18 (0.9%) |
| **Starter Pet** | Agree | 593 (16.1%) | 180 (28%) | 251 (11.6%) | 468 (23.5%) |
|  | Partly agree | 915 (24.8%) | 222 (34.6%) | 455 (20.9%) | 618 (31.1%) |
|  | Partly disagree | 704 (19.1%) | 99 (15.4%) | 388 (17.9%) | 386 (19.4%) |
|  | Disagree | 1410 (38.2%) | 128 (19.9%) | 1040 (47.9%) | 477 (24%) |
|  | Uncertain | 71 (1.9%) | 13 (2.0%) | 38 (1.7%) | 39 (2.0%) |
| **WTP** | 0 (would ask for euthanasia) | 490 (13.3%) | 177 (27.6%) | 115 (5.3%) | 520 (26.2%) |
|  | 1-999 DKK | 242 (6.6%) | 72 (11.2%) | 82 (3.8%) | 218 (11%) |
|  | 1000-1999 DKK | 647 (17.5%) | 134 (20.9%) | 311 (14.3%) | 429 (21.6%) |
|  | 2000-4999 DKK | 922 (25.0%) | 114 (17.8%) | 635 (29.2%) | 372 (18.7%) |
|  | 5000-9999 DKK | 486 (13.2%) | 33 (5.1%) | 386 (17.8%) | 128 (6.4%) |
|  | 10000-29999 DKK | 186 (5%) | 8 (1.2%) | 166 (7.6%) | 25 (1.3%) |
|  | 30000-59999 DKK | 43 (1.2%) | 0 (0.0%) | 38 (1.7%) | 5 (0.3%) |
|  | 60000-99999 DKK | 24 (0.6%) | 4 (0.6%) | 23 (1.1%) | 5 (0.3%) |
|  | > 100000 DKK | 107 (2.9%) | 7 (1.1%) | 99 (4.6%) | 13 (0.7%) |
|  | Uncertain | 546 (14.8%) | 93 (14.5%) | 317 (14.6%) | 273 (13.7%) |

**Table S4** (continued)
Responsibility: The main person responsible for the care of the rabbit in the household; Starter Pet: Owner-opinion on the suitability of rabbits as first time companion animals for children; WTP: Willingness-To-Pay for veterinary treatment.
DKK: Danish crowns (1 DKK = 0.13 EUR; exchange rate per Jan. 2023).

**Survey I questionnaire**

Translated version (from Danish to English) of the questionnaire for Survey I. Note that not all questions in the original survey are included in this version of the questionnaire (questions relating to other species) and not all questions included here are utilised in the current study. Moreover, some questions were only visible to the respondent dependent on previous responses. Multiple choice questions, where the respondent could choose more than one answer, are indicated by the text “Feel free to choose more than one answer” below the question.

[Introduction] Thank you for answering our survey on attitudes towards and keeping of pets and hobby animals. The study is carried out by researchers at the University of Copenhagen. The results of the survey will be used for research.

Thank you in advance for your help.

[Questionnaire]

**How is your household composed?**

- I live alone
- I live with others (e.g. partner/spouse, children or friend)
- Other (e.g. at a dormitory)

**What gender do you identify as?**

- Male
- Female
- Other
- Prefer not to say

**What is your age?**
___ years

Pets and hobby animals are often kept in the home, but we would also like to hear about animals that are not kept in the home (e.g. horses). On the other hand, we are not here interested here in typical agricultural and production animals such as chicken and sheep.

**Do you have one or more pets/hobby animals?**

- No
- Yes

**What pets/hobby animals do you have and how many?**

- None
- 1
- 2
- 3
- More than 4

[…] Now we want to ask about your rabbit.

If you have more than one rabbit, you should in the following questions only answer for the rabbit whose name starts with the first letter in the alphabet.

**What sex is the rabbit?**

- Male
- Female
- Don’t know

**How old is the rabbit?**Please enter the age in whole years
___ years

**Is the rabbit spayed or neutered?**

- Yes
- No
- Don’t know

**How did you acquire the rabbit?**

- I/we have bred it ourselves
- Private home
- Animal shelter or relocation home
- Breeder
- Pet store
- Imported from another country
- Other
- Don't know

**How did you get in touch with those that you got the rabbit from?**Choose the category that fits best.

- Via websites on the internet (e.g. ‘DBA’ or ‘Gul & Gratis’)
- Newspapers/magazines
- Via friends and acquaintances
- Via an animal shelter
- Via other people who have rabbits
- Via social media (including Facebook groups)
- Other
- Don't know

**For what purpose did you acquire the rabbit?**

Feel free to choose more than one answer

- Companion animal - acquired for an adult (over 17 years)
- Companion animal- acquired for child or teenager (up to 17 years)
- Breeding
- Exhibition
- Show jumping
- Meat/fur
- Other
- Don't know

**Do you mainly keep the rabbit indoors or outdoors?**

- Mainly indoors
- Mainly outdoors
- Both indoors and outdoors (e.g. depending on the season)
- Don’t know

**How do you mainly keep the rabbit in your residence?**

- In cage with no access to other area
- In a cage with a certain degree of access to a restricted area (e.g. rabbit run/aviary)
- In a cage with some access to all/most of the home or garden
- In a restricted area with some access to the rest of the home or the garden
- In a restricted area all the time
- Completely free roam in all/most of the home or garden
- Other
- Don’t know

**Which of the following does the rabbit have access to on a daily basis?** [multiple choice]

Feel free to choose more than one answer

- Digging opportunities (e.g. digging box or substrate in the garden)
- Gnawing opportunities (e.g. branches, cardboard boxes, gnawing toys)
- Hiding places (e.g. cave/house in the base or cardboard box)
- Toys/other activation (e.g. problem-solving toys)
- Space to run and jump around (outside of the cage)
- Human contact/supervision
- Spending time with other rabbit(s).
- Don’t know

**How often is the rabbit vaccinated?**

- Each year
- Every two years
- Every third year
- Rarer
- Never
- Do not know

**How often do you take the rabbit to the vet?**You must not include vaccination visits.

- More than once a year
- Once a year
- Every two years
- Rarer
- Never been to the vet
- Do not know

**Is the rabbit marked?** [multiple choice]

Feel free to choose more than one answer

- Ear tagged but not registered
- Earmarked and registered
- Chip-marked but not registered
- Chip-marked and registered
- No
- Don't want to answer
- Don’t know

**What insurances and health schemes are there on the rabbit?** [multiple choice]

Feel free to choose more than one answer

- Health insurance
- Health scheme at the vet
- Other
- None
- Don't want to answer
- Don’t know

**What does it take for you to take the rabbit to the vet?**Do not count vaccinations and routine health checks.

- Do not take the rabbit to the vet
- That there are signs of serious illness
- That there are signs of less serious illness
- Other
- Don't want to answer
- Don’t know

**Has the rabbit had the following illnesses or health problems within the past year?** [multiple choice]

Feel free to choose more than one answer

- A short-term illness (e.g. infection)
- Prolonged/chronic illness
- No, none of these
- Don’t know

**Has the rabbit had any of the following behavioural problems within the past year?** [multiple choice]
 Feel free to choose more than one answer

- Digs into hard surfaces (e.g. the floor) or bites the cage
- Is distinctly inactive (in the evening and morning hours) and seems uninterested in its surroundings
- Shows aggressive behaviour towards, or fear of, other rabbits/other animals in the home
- Shows aggressive behaviour towards or fear of any of you
- Stomps excessively, hides or isolates itself a lot of the time
- Grooming that leads to bald spots
- Other behavioural problems
- None of these
- Don’t know

**Who in the household…**

- was most eager for getting the rabbit
  - All (or most) in the home
  - Myself
  - My partner/spouse
  - My children
  - My parents
  - Others
  - Don’t know
- is most attached to the rabbit
  - All (or most) in the home
  - Myself
  - My partner/spouse
  - My children
  - My parents
  - Others
  - Don’t know
- spends most time on activities with the rabbit
  - All (or most) in the home
  - Myself
  - My partner/spouse
  - My children
  - My parents
  - Others
  - Don’t know
- most often feeds the rabbit
  - All (or most) in the home
  - Myself
  - My partner/spouse
  - My children
  - My parents
  - Others
  - Don’t know

**Survey II questionnaire**

Translated version (from Danish to English) of the questionnaire for Survey II. Note that not all questions in this version of the questionnaire are utilised in the current study and that some questions were only visible to the respondent dependent on previous responses (questions related to rabbits kept for other purposes than companion animals and others sections concerning the Lexington Attachment to Pets Scale (LAPS), mental abilities of rabbits, and behavioural problems, among others, are also excluded from this version to limit the length of the text). Multiple choice questions, where the respondent could choose more than one answer, are indicated by the text “Feel free to choose more than one answer” below the question.

[Introduction]

**Questionnaire:** Keeping and caring for rabbits - a survey on the conditions for domestic rabbits in Denmark

This questionnaire is part of a larger study on the conditions for domestic rabbits in Denmark, as well as the relationship of rabbit owners to rabbits.

The questionnaire is aimed at people over 18 years living in a household in Denmark where rabbits are kept, either as companion animals (pets) or for breeding/other hobby purposes.

The questionnaire is not aimed at farms with commercial breeding of rabbits for slaughter.

Note that the questionnaire must only be answered once per household.

It takes approx. 10 - 15 min. to complete the survey.

The results of the study will be published in a report to the Danish Veterinary and Food Administration, in a scientific article and possible derivative articles.

Participation in the questionnaire is voluntary and your answers will remain anonymous. By answering the questionnaire, you give permission for your answer to be used for the above purposes. Along the way, you will have the opportunity to withdraw your answer - to do this, simply close the browser window with the questionnaire. However, after pressing "Finish" at the end of the questionnaire, you can no longer withdraw your answer.

Note that responses are not processed individually and will only be included in an overall collection of data, whereby results cannot be traced back to individual responses.

By pressing "Next" you consent to participate in the survey.

The project is carried out by the Department of Veterinary and Animal Science, University of Copenhagen, Grønnegårdsvej 8, 1870 Frederiksberg C, and is financed by the Knowledge Center for Animal Welfare, Danish Veterinary and Food Administration. (Project leader: Professor Björn Forkman).

Inquiries regarding the questionnaire should be directed to Cecilie Ravn Skovlund, industrial PhD fellow: wqg189@sund.ku.dk

Thank you for your interest in the questionnaire. Press "Next" to access the questions.

[Questionnaire]

**Do you keep one or more rabbits, either as pets or for other purposes?***

This refers to rabbits in the home as well as rabbits kept for hobby purposes and hobby breeding.

Rabbits that are bred for slaughter at hobby level and/or self-sufficiency are also included, except for rabbits that are part of commercial rabbit meat production.

- Yes
- No

**(This questionnaire is aimed at people who keep rabbits. Since you have indicated that you do not keep rabbits, the questionnaire closes. Thank you for your interest.*

*Finish by clicking 'Finish' below.)*

**What is your age?****To participate in this survey, you must be 18 years of age.

_________

***(This questionnaire is aimed at people of the age of 18 who keep rabbits. Since you have indicated that you are under 18 years of age, the questionnaire closes. Thank you for your interest.*

*Finish by clicking 'Finish' below.)*

**Where in the country do you live?**

ZIP code to be filled in voluntarily

_________

**What type of housing do you live in?**

Please choose the one that suits you best

- House/terrace house in a residential area
- Apartment
- House in the country side/previous farm
- Active farming
- Other

**What gender do you identify as?**

- Female
- Male
- Other
- Don't want to answer

**What is your highest completed education?**

- Primary school
- Secondary education
- Vocational education
- Labour market/specialist worker training
- Short higher education
- Intermediate higher education
- Long higher education
- Other education
- Don't want to answer

**How many adults (18 years and over) are there in your household, including yourself?**

Number of adults (including yourself): _________

**How many children (under the age of 18) are there in your household?**

Number of children (under 18): _________

**Do you keep rabbits as companion animals or for other purposes?**

“Companion animal" refers to "pet".

Rabbit keeping for other purposes refers to rabbits that are kept or bred in relation to a function other than family animals (e.g. rabbits for breeding, fancy rabbits or meat).

If you have both types of rabbit keepings, or have rabbits that are part of both keepings at the same time, please choose the category in which you have the most rabbits.

In this questionnaire, you only have the option of entering information about one type of rabbit keeping.

- Companion animal
- Other purpose (e.g. breeding/hobby breeding or other purpose)

**What is the purpose of the rabbit keeping/breeding?** ***

If you are keeping rabbits for several purposes, or you have several types of rabbit keepings, please choose the category in which you have the most rabbits.

- Breeding/keeping of meat rabbits
- Breeding/keeping rabbits for breeding
- Breeding/keeping rabbits for exhibition (fancy rabbits)
- Breeding/keeping rabbits for show jumping
- Breeding/keeping of angora rabbits for wool
- Breeding/keeping rabbits for fur/skin
- Breeding/keeping rabbits for animal feed
- Breeding/keeping rabbits for training hunting dogs
- Other (specify other type if necessary)

***(*The remaining questions aimed at persons keeping/breeding rabbits for other purposes are excluded from the remainder of the translated questionnaire*)

**How many rabbits do you have as pets?**

In the following questions, please answer for the rabbit whose first letter in the name comes first in the alphabet.

If you have several rabbits with the same first letter in the name, the next letter in the name is taken, etc.

**What is the rabbit's age?**

- Less than 1 year
- 1 year
- 2 years
- 3 years
- 4 years
- 5 years
- 6 years
- 7 years
- 8 years
- 9 years
- 10 years
- 11 years
- 12 years
- 13 years or older
- Don’t know

**How many years have you had the rabbit?**

- Less than 1 year
- 1 year
- 2 years
- 3 years
- 4 years
- 5 years
- 6 years
- 7 years
- 8 years
- 9 years
- 10 years
- 11 years
- 12 years
- 13 years or older
- Don’t know

**What is the gender of the rabbit?**

- Male
- Female
- Don’t know

**Is the rabbit neutered or spayed?**

- Yes
- No
- Don’t know

**What breed is the rabbit?** *(Danish breed names are not translated here. The breed list is adapted from the list by the Danish Rabbit Breeder Association (DKAF) (Danish Rabbit Breeder Association, n.d.); “Other breed” therefore represents breeds not on the DKAF list.)*

- [Response options] Alaska; Angora; Beige; Belgisk Hare; Belgisk kæmpe; Beveren; Blandingsrace; Blå Wiener; Bourgogne; Californian; Cashmere Vædder; Deilenaar; Dværghare; Dværgschecke; Dværgvædder; Dværgvædder, rex; Dværgvædder, satin; Engelsk Schecke; Engelsk Vædder; Fransk Vædder; Fuchs; Gouwenaar; Hermelin; Hermelin, angora; Hermelin, fuchs; Hermelin, rex; Hermelin, satin; Hollænder; Hotot; Hvid Land; Isabella; Jamora; Japaner; Lille Chinchilla; Lille Egern; Lille Havana; Lille Rex; Lille Satin; Lille Sølv; Lille Tysk Schecke; Lille Tysk Vædder; Lille Wiener; Lux; Løvehoved; Løvehoved dværgvædder; Marburger Egern; Mecklenburger Schecke; Meissner Vædder; New Zealand Red; New Zealand White; Orange; Perle Egern; Rex; Rhinsk Schecke; Rhön; Russer; Sachsengold; Sallander; Satin; Satinangora; Schwarzgrannen; Stor Chinchilla; Stor Havana; Stor Marburger Egern; Stor Sølv; Stor Zobel; Svensk Pels; Tan; Thyringer; Trønder; Tysk Kæmpeschecke; White; Wiener; Zobel; Ørestad; Other breed; Don’t know

**Who in the household is the rabbit acquired for?**

- Myself/other adult (18 years or over)
- Child 0-5 years
- Child 6-11 years
- Child 12-17 years
- Other

**Who in the household is primarily responsible for the daily care of the rabbit?**

- Myself
- Cohabitant/other adult (18 years or over)
- Child 0-5 years
- Child 6-11 years
- Child 12-17 years
- The responsibility is equally distributed in the household
- Other

**Where was the rabbit acquired from?**

- I/we bred it myself/ourself
- From a private home
- Shelter or relocation home
- A breeder
- A pet store/garden centre
- Flea market or yard sale
- Got the rabbit as a gift
- Found as a stray
- Other
- Don’t know

**Is the rabbit housed with other rabbits?**

- Yes, all the time
- Yes, but only at certain times of the day
- No, but the rabbit has the possibility of tactile contact with other rabbits (e.g. through a wire fence)
- No, but the rabbit has visual contact (without the possibility of touching) with another rabbit(s).
- No, the rabbit is kept alone

**What is the reason for keeping the rabbit alone or having a single rabbit?**

Feel free to choose more than one answer

- The rabbit has become alone (e.g. due to death of partner rabbit) and I don't want any more rabbits
- Other
- It is difficult/not possible to keep the rabbit with other rabbits (due to behavioural or health reasons)
- Rabbits should generally be alone/do best alone
- Having more rabbits requires more resources (e.g. space, time and money)
- I am worried that the rabbit will become less contact-seeking/tame
- Because I don't want to neuter/spay the rabbits
- I am currently looking for another rabbit, for the rabbit
- Rabbits thrive fine alone
- I don't want to/don't have the conditions to bond rabbits (e.g. lack of space)
- Don’t know

**If 'Other', what other reason is there for you to keep the rabbit alone/a single rabbit?**

_________

**What is the reason for keeping the rabbit with another rabbit(s)?**

Feel free to choose more than one answer

- Rabbits should generally be with/thrive better with other rabbits
- Other
- So that the rabbits have the opportunity to socialise with each other
- Because I found an extra rabbit that I would like to keep/provide a home for
- Because I like rabbits and want to have more of them
- So that I can breed them
- So that I don't have to interact nearly as much with the rabbits (they entertain each other)
- Don’t know

**If 'Other', what other reason is there for you keeping the rabbit with another rabbit(s)?**

_________

**Is the other rabbit in the pair, or the other rabbits in the group, neutered or spayed?**

- Yes, the other(s) are neutered/spayed
- Only some of the others are neutered/spayed
- No
- Don’t know

**Is the rabbit housed together with other animals?**

Feel free to choose more than one answer

- Dog
- Cat
- Rodents (e.g. guinea pig, hamster or rat)
- Chickens
- Other bird
- Reptile or amphibian
- Other animal
- No

**Do you keep the rabbit as an indoor or outdoor rabbit?**

- Exclusively as an indoor rabbit
- Mainly as an indoor rabbit
- Exclusively as an outdoor rabbit
- Mainly as an outdoor rabbit
- Both as an indoor and outdoor rabbit (equally distributed, e.g. depending on the season)

**How often does the rabbit have access to an outdoor area (when the weather permits it)?**

- All the time/several times a day
- Daily
- Several times a week
- Weekly
- Monthly
- Rarer
- Never

**Does the rabbit have access to an area that protects against wind, weather and the cold when it is kept (has its base) outdoors?**

- Yes
- No
- Not relevant
- Don’t know

**How do you mainly keep the rabbit in your residence?**

If the rabbit has a cage that is open 24/7, it is not considered a cage in the options below.

Please choose the option that suits you best

- Exclusively in a cage, without access to other areas
- In a cage with access to a restricted area (e.g. rabbit run/aviary) at certain times of the day (e.g. when I am at home/awake)
- In a cage with access to all/most of the home or garden at certain times
- In a restricted area all the time (e.g. rabbit run/aviary)
- In a restricted area with access to the rest of the home or the garden at certain times
- Exclusively as free roaming in all/most of the home or garden

**What is the reason for keeping the rabbit in a cage?**

Feel free to choose more than one answer

- The rabbit is not clean, is noisy or destroys things (e.g. gnaws on furniture)
- Rabbits do not need a lot of space
- So that the rabbit stays clean/doesn't get dirty (e.g. an angora rabbit)
- The rabbit is aggressive or fearful (e.g. towards you or other rabbits/animals)
- Other
- To avoid unwanted mating
- So that the rabbit can be outside without running away
- For the rabbit's own safety (e.g. due to electrical cords or predators)
- Don’t know

**If 'Other', what other reason is there for keeping the rabbit in a cage?**

_________

**What is the reason for keeping the rabbit partially or completely free roam?**

Feel free to choose more than one answer

- Rabbits need space to thrive
- Rabbit cages take up too much space/don't look nice
- I am generally against keeping animals in cages
- I get more out of keeping the rabbit when it runs free (e.g. socialising)
- The rabbit was previously in a cage but did not thrive (e.g. it bit the bars or was cage-aggressive)
- Other
- Don’t know

**If 'Other', what other reason is there for keeping the rabbit partially or completely free range?**

_________

**What are the dimensions of the cage?**

Both length, width (m) and the total area (m2) are asked for, in order to account of different cage shapes.

If you do not wish to answer one or more, you can simply leave the fields blank.

Longest part of the cage, in m (meters): _________

Widest part of the cage, in m (meters): _________

Total area, in m2 (square meters): _________

Total height, in m (meters): _________

Number of floors (incl. the cage bottom): _________

**How many rabbits are housed together in the cage?** _________

**How large is the area of ​​the free roam area (e.g. run, house or garden)?**

Please specify in m2 (square meters).

If you don't want to answer, you can simply press "Next".

 _________

**How many rabbits are housed together in the free roam area?:** _________

**How many hours in a typical day does the rabbit have the opportunity to get out of the cage?**

- The rabbit does not get out of the cage on a typical day
- Under 1 hour
- 1 - 3 hours
- 4 - 6 hours
- 7 - 9 hours
- 10 - 12 hours
- Over 12 hours
- Don’t know

**What time of the day does the rabbit typically have the opportunity to get out of the cage to a larger area?**

Feel free to choose more than one answer

- In the morning
- In the forenoon
- In the afternoon
- In the evening
- At night
- Don’t know

**How often does the rabbit have access to the following:**

- Digging opportunities (e.g. digging box or a diggable substrate such as soil)
  - Always
  - Sometimes
  - Never
- Gnawing opportunities (e.g. branches, cardboard boxes, gnawing toys)
  - Always
  - Sometimes
  - Never
- Hiding places (e.g. cave/house, tunnel or cardboard box)
  - Always
  - Sometimes
  - Never
- Problem solving toys
  - Always
  - Sometimes
  - Never
- Other toys/other activation
  - Always
  - Sometimes
  - Never
- Elevated platform(s) for the rabbit to jump on
  - Always
  - Sometimes
  - Never

**How often does the rabbit get the following:**

- Hay or grass
  - Available all the time (ad libitum)
  - Daily
  - Several times a week
  - Weekly
  - Monthly
  - Rarer
  - Never
- Homogenous pellets for rabbits
  - Available all the time (ad libitum)
  - Daily
  - Several times a week
  - Weekly
  - Monthly
  - Rarer
  - Never
- Rodent or muesli mix
  - Available all the time (ad libitum)
  - Daily
  - Several times a week
  - Weekly
  - Monthly
  - Rarer
  - Never
- Leafy greens (e.g. dandelions or herbs)
  - Available all the time (ad libitum)
  - Daily
  - Several times a week
  - Weekly
  - Monthly
  - Rarer
  - Never
- Other vegetables (e.g. vegetables, root vegetables or fruit)
  - Available all the time (ad libitum)
  - Daily
  - Several times a week
  - Weekly
  - Monthly
  - Rarer
  - Never
- Branches or roots
  - Available all the time (ad libitum)
  - Daily
  - Several times a week
  - Weekly
  - Monthly
  - Rarer
  - Never

**What type of water source does the rabbit have access to?**

Feel free to choose more than one answer

- Drinking bottle/drinking nipple
- Water bowl, water fountain or other open body of water
- Natural water source (e.g. lake/river)
- Other

**How often is the rabbit's water changed?**

- Several times a day
- Daily
- Several times a week
- Weekly
- Monthly
- Rarer
- Never
- Not relevant (e.g. water via lake/river)

**Is the rabbit regularly seen by a veterinarian for health check-ups/preventive treatment?**

1. Yes, more than once a year
2. Yes, once a year
3. Yes, every other year
4. Yes, less often
5. No, only in case of acute illness
6. No, not even in case of acute illness
7. Don’t know

**Is the rabbit health insured?**

- Yes
- No
- Don’t know

**Is the rabbit regularly vaccinated?**

- Yes, every year
- Yes, every other year
- Yes, every three years
- Yes, less often
- No
- Don’t know

**Is the rabbit marked and/or registered?**

- Earmarked
- Earmarked and registered
- Chip-marked
- Chip-marked and registered
- No
- Don’t know

**How long do you approx. spend each day being close to, care for and/or be with the rabbit?**

- I don't see the rabbit every day
- 5 minutes or less
- 6-15 minutes
- 16-30 minutes
- 31-45 minutes
- 46-60 minutes
- 1-2 hours
- 2-3 hours
- More than 3 hours
- Don’t know

[Not health insured] **Imagine that the rabbit is seriously ill and suffering. The vet has said that the rabbit can recover completely if an operation is carried out. If the operation is not carried out, the vet recommends that the rabbit be euthanised.**

**How much money would you be prepared to pay for such an operation?**

- I would forgo surgery and ask for euthanasia
- Up to DKK 999
- 1000-1999 DKK
- 2000-4999 DKK
- 5000-9999 DKK
- 10000-29999 DKK
- 30000-59999 DKK
- 60000-99999 DKK
- DKK 100,000 or more
- Don’t know

[Health insured] **Imagine that the rabbit is seriously ill and suffering. The vet has said that the rabbit can recover completely if an operation is carried out. If the operation is not carried out, the vet recommends that the rabbit be euthanised. Unfortunately, your insurance does not cover the entire amount needed for the operation.**

**How much money would you be prepared to pay on top of the sum insured for such an operation?**

- I would forgo surgery and ask for euthanasia
- Up to DKK 999
- 1000-1999 DKK
- 2000-4999 DKK
- 5000-9999 DKK
- 10000-29999 DKK
- 30000-59999 DKK
- 60000-99999 DKK
- DKK 100,000 or more
- Don’t know

**Do you also keep rabbits for a different purpose than the one you have entered for so far?**

Feel free to choose more than one answer

- Rabbit as a companion animal
- Breeding/keeping of meat rabbits
- Breeding/keeping rabbits for breeding
- Breeding/keeping rabbits for exhibition (fancy rabbits)
- Breeding/keeping rabbits for show jumping
- Breeding/keeping of angora rabbits for wool
- Breeding/keeping rabbits for fur/skin
- Breeding/keeping rabbits for animal feed
- Breeding rabbits for training hunting dogs
- Other (specify other type if applicable)
- No

**Why do you in general have a rabbit as a companion animal?**

Feel free to choose more than one answer

- It does not cost a lot of money to keep rabbits
- Rabbits are a good starter pet (e.g. for children)
- I am allergic to other animals
- Rabbits are good pets/good company
- Rabbits can be used for several things besides pets (e.g. for exhibition, jumping, meat)
- To help a rabbit/rabbits
- Rabbits are easy to keep/easier to keep than other animals (e.g. dogs)
- Other
- I regret getting a rabbit
- Others in the household wanted rabbit
- Rabbits are cute/pretty
- Don’t know

**If 'Other', please specify other reason why you have a rabbit as a pet**_________

**You may specify the reason for regretting getting a rabbit as a pet** _________

**How old was your previous rabbit at death/euthanasia?**

- Less than a year
- 1 year
- 2 years
- 3 years
- 4 years
- 5 years
- 6 years
- 7 years
- 8 years
- 9 years
- 10 years
- 11 years
- 12 years
- 13 years or older
- Haven't experienced losing a rabbit
- Don’t know

**How many years have you kept rabbits in total?**
 _________

**Did you have a rabbit as a child (0-17 years)?**

- Yes
- No
- Don’t know

**How much do you agree with the following statements about the suitability of rabbits as pets?**

- Rabbits are suitable for children
  - Disagree
  - Partly disagree
  - Partially agree
  - Agree
  - Don’t know
- Rabbits are suitable as starter pets for children
  - Disagree
  - Partly disagree
  - Partially agree
  - Agree
  - Don’t know
- Rabbits are only suitable for adults
  - Disagree
  - Partly disagree
  - Partially agree
  - Agree
  - Don’t know
- Rabbits are not suitable as pets
  - Disagree
  - Partly disagree
  - Partially agree
  - Agree
  - Don’t know
- Rabbits are only suitable for adults with extensive knowledge of the species
  - Disagree
  - Partly disagree
  - Partially agree
  - Agree
  - Don’t know
- Rabbits are suitable for children with the help/support of a responsible adult
  - Disagree
  - Partly disagree
  - Partially agree
  - Agree
  - Don’t know

**Thank you very much for your participation!**

**Your response aids in generating new knowledge that is important for mapping, and potentially improving, the conditions for domestic rabbits in Denmark, and which may benefit both rabbits and rabbit owners.**

**Submit your answer by clicking 'Finish' below.**
